# A Modular Approach to Triazole-Containing Chemical Inducers of Dimerisation for Yeast Three-Hybrid Screening

**DOI:** 10.3390/molecules180911639

**Published:** 2013-09-23

**Authors:** Fanny Tran, Anahi V. Odell, Gary E. Ward, Nicholas J. Westwood

**Affiliations:** 1 School of Chemistry and Biomolecular Sciences Research Complex, University of St Andrews and EaStCHEM, North Haugh, St Andrews, Fife, Scotland KY16 9ST, UK; 2 Department of Microbiology and Molecular Genetics, 316 Stafford Hall, University of Vermont, 95 Carrigan Drive, Burlington, VT 05405, USA

**Keywords:** click chemistry, yeast three-hybrid approach, CIDs

## Abstract

The yeast three-hybrid (Y3H) approach shows considerable promise for the unbiased identification of novel small molecule-protein interactions. In recent years, it has been successfully used to link a number of bioactive molecules to novel protein binding partners. However despite its potential importance as a protein target identification method, the Y3H technique has not yet been widely adopted, in part due to the challenges associated with the synthesis of the complex chemical inducers of dimerisation (CIDs). The development of a modular approach using potentially “off the shelf” synthetic components was achieved and allowed the synthesis of a family of four triazole-containing CIDs, **MTX-Cmpd2.2-2.5**. These CIDs were then compared using the Y3H approach with three of them giving a strong positive interaction with a known target of compound **2**, TgCDPK1. These results showed that the modular nature of our synthetic strategy may help to overcome the challenges currently encountered with CID synthesis and should contribute to the Y3H approach reaching its full potential as an unbiased target identification strategy.

## 1. Introduction

**Figure 1:**
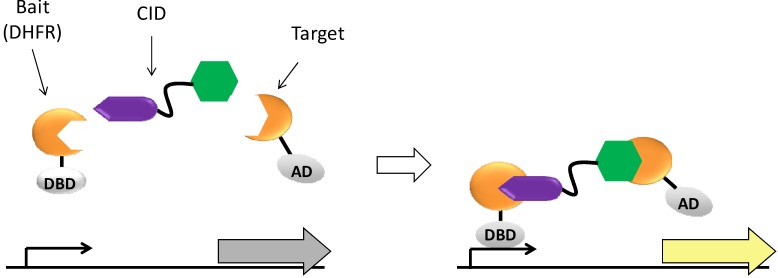
Schematic representation of the Y3H system used in this study showing the ternary complex between the chemical inducer of dimerization (CID) and the two fusion proteins containing the activation and DNA-binding domains of the transcription factor (AD and DBD, respectively). The CID consists of: (i) methotrexate (shown in purple), which binds to the dihydrofolate reductase DHFR-DBD fusion protein; (ii) a flexible linker unit (black line) and (iii) the bioactive molecule of interest (blue hexagon), which binds to the target protein-AD fusion. Successful formation of the ternary complex results in expression of the reporter genes (e.g., *LEU2*) that enables the yeast cell to grow in the absence of the amino acid.

The yeast three-hybrid (Y3H) approach shows considerable promise for the identification of novel small molecule-protein interactions [1,2,3,4,5,6,7,8,9,10,11,12,13,14,15,16,17]. In recent years, this unbiased approach has linked a number of bioactive molecules to novel protein binding partners generating new biological hypotheses that have been investigated further using alternative experimental techniques [2,3,6,9,13]. For example, Johnsson discovered using Y3H that sulfasalazine, a drug used against inflammatory bowel disease, inhibits tetrahydrobiopterin biosynthesis and consequently nitric oxide production through its interaction with sepiapterin reductase [6]. More recently, Cornish used Y3H to identify PDE6D as a novel protein target of anecortave acetate, an intraocular pressure-lowering agent used in the treatment of glaucoma [18]. At the heart of the Y3H approach is an ingenious system to screen for potential binding proteins [13,15,19,20,21,22]. The screen is carried out in yeast cells as the successful formation of a ternary complex consisting of the *bait*, the *target protein* and a *chemical inducer of dimerisation* (CID) results in yeast cell growth *via* the activation of the required yeast reporter gene (see Figure 1 for details). The availability of the CID, a compound that contains the bioactive molecule of interest, a linker unit and typically methotrexate (MTX) is essential.

**Figure 2:**
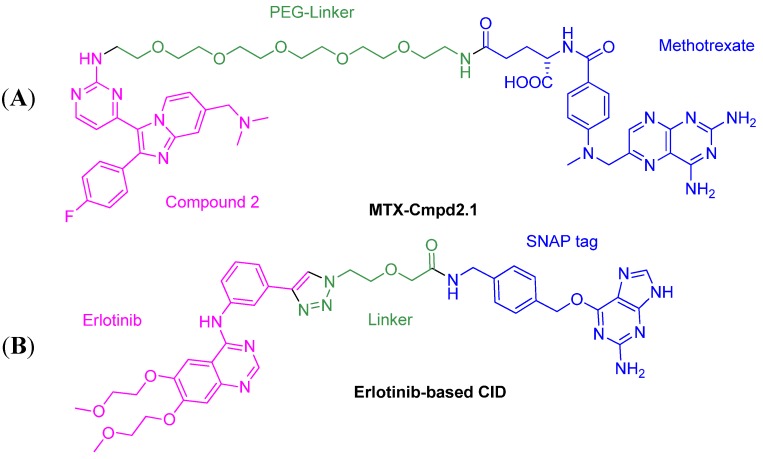
Structures of (**A**) CID **MTX-Cmpd2.1** bearing a PEG linker and (**B**) the erlotinib-based CID as described by Chidley *et al*. [6].

Despite its potential importance as a protein target identification method, the Y3H technique has not yet been widely adopted in part due to the challenges associated with the synthesis of the high molecular weight (>1,000 Da) and relatively complicated CIDs. For example, we recently reported the use of Y3H to identify Tg*BRADIN* as a target of compound **2** [23]. Tg*BRADIN* is a previously unknown negative regulator of the apicomplexan parasite *Toxoplasma gondii*’s tachyzoite to bradyzoite differentiation pathway. In that study we used a CID that contained a PEG linker unit **MTX-Cmpd2.1** (Figure 2A), in line with the majority of the existing literature [3,6,11,24]. **MTX-Cmpd2.1** was prepared in a total of 21 steps with seven of the steps being required just to make the linker unit.

In the course of our Y3H project, we became interested in preparing additional CIDs that contained the same bioactive molecule (compound **2**) and methotrexate but that differed in the length of the linker. The question of whether linker length affects the ability of a CID to dimerise proteins has been previously examined in three-hybrid systems, but with conflicting results [19,25,26]. For example, the length of the linker appeared to have no effect on the dimerisation of FK506-binding proteins leading to signal transduction [26]. Similarly, Cornish saw high levels of transcriptional activation induced in the Y3H system using dihydrofolate reductase and the glucocorticoid receptor, regardless of CID structure and linker length [19]. In contrast, Amara *et al.* found dramatic effects of the linker length when comparing the ability of a series of four CIDs to dimerize tandem FKBP fused either to Fas proteins leading to apoptosis or to the domains of a transcription factor allowing reporter gene transcription [25]. The discrepancies between these studies might be explained by the relative strength of the interactions, the distance between the two proteins required to exert their effects or steric hindrance imposed by the proteins. Unfortunately these variables are extremely difficult to anticipate when designing a CID for use in Y3H-based drug target identification, suggesting that the development of a versatile synthetic strategy to enable the rapid generation of related CIDs (families of CIDs) may be advantageous. We therefore decided to establish a route to families of compound **2**-based CIDs that differed only in the linker unit using potentially “off the shelf” reagents.

**Scheme 1:**
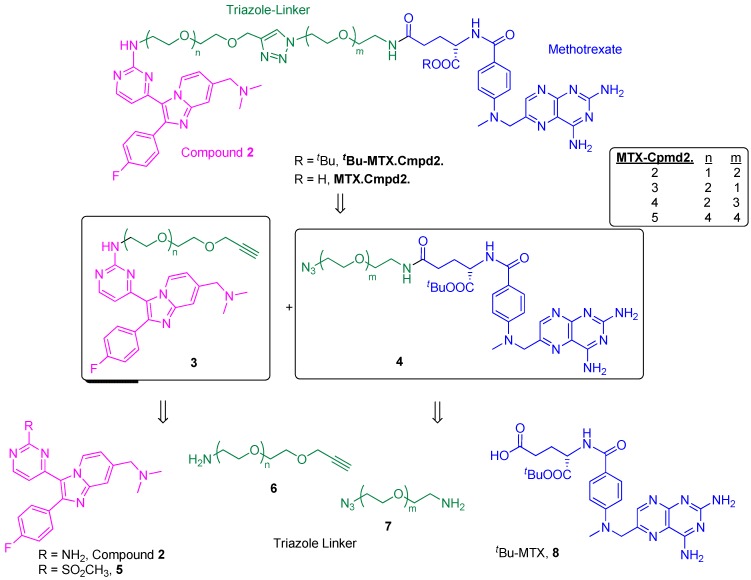
Modular approach to **MTX-Cmpd2** CIDs enabling components to be mixed and matched as required.

In 2011, Johnsson reported the synthesis of a 1,2,3-triazole-containing CID based on the clinically approved drug erlotinib (Figure 2B). Use of this CID in Y3H enabled the identification of the binding partner oxysterol-binding protein-related protein 7, the first non-kinase target identified for this drug [6]. Based on this literature precedent, we investigated the use of the copper-catalysed Hüisgen 1,3-dipolar cycloaddition reaction between an azide and an alkyne to generate CIDs in a modular fashion. Here we report the application of this approach to the rapid synthesis of a family of four CIDs with varying linker lengths, **MTX-Cmpd2.2-2.5** (Scheme 1). A comparison of our CIDs **MTX-Cmpd2.1-2.5** in the Y3H approach is also described. Significantly, lower background growth was observed with some of our new triazole-containing CIDs than with our original PEG-containing CID, **MTX-Cmpd2.1**.

## 2. Results and Discussion

An outline of the modular approach that was adopted to target **MTX-Cmpd2** CIDs is shown in Scheme 1. The planned synthesis involved the coupling of two key components: the *compound* **2***-based alkyne* **3** (where n is variable) and the *^t^Bu-MTX-azide* **4** (where m is variable). Components **3** and **4** could be accessed by the synthesis of a precursor to compounds **2** and **5**, various PEG-based linker units **6** and **7** and *tert*-butyl methotrexate (*^t^*Bu-MTX, **8** [23]) (Scheme 1).

**Figure 3:**
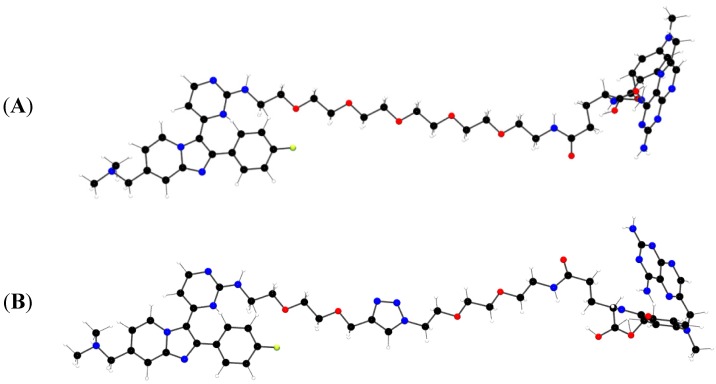
Examples of predicted 3D conformations of (**A**) **MTX-Cmpd2.1** and (**B**) **MTX-Cmpd2.2**.

To determine whether the synthetic plan would work and to assess whether a triazole-containing linker could be tolerated in this system, initial studies focused on the synthesis of **MTX-Cmpd2.2** (n = 1 and m = 2), which was close in structure to the original CID **MTX-Cmpd2.1**. 3D Representations of **MTX-Cmpd2.1** and **MTX-Cmpd2.2** (generated using low level computational methods inspired by the report of Lu *et al*. [27]) suggested that the planned change in the linker system would give CID **MTX-Cmpd2.2**, with little significant impact on the overall length of the CID despite the fact that the linker unit in **MTX-Cmpd2.2** contains an additional atom. Again in agreement with the work of Lu *et al.* [27], the predicted extended conformation of **MTX-Cmpd2.1** was linear, whereas that for **MTX-Cmpd2.2** was not (Figure 3).

### 2.1. Comparison of **MTX-Cmpd2.1** (PEG Linker) and **MTX-Cmpd2.2** (Triazole Linker)

#### 2.1.1. Synthesis of the Compound **2**-based Alkynes **3**

**Scheme 2:**
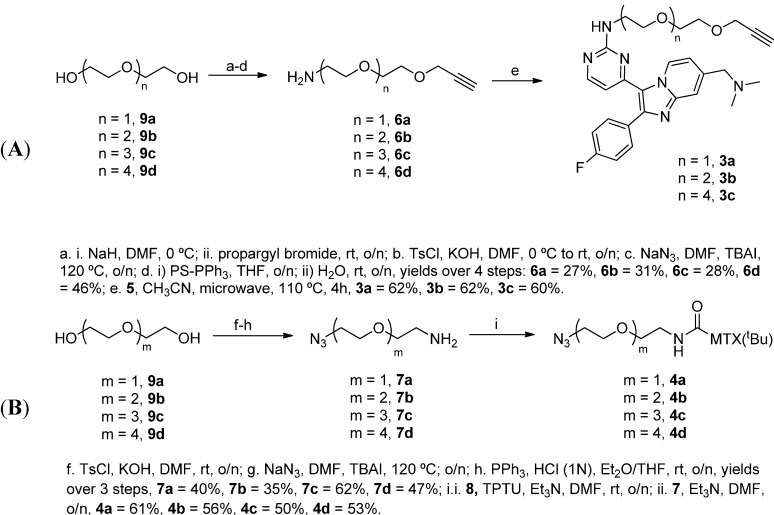
Synthesis of (**A**) Compound**2** based alkyne **3** and (**B**) *^t^*Bu-MTX azide **4**.

The required sulfone **5** was successfully synthesised in gram quantities as reported previously by us and others [9,23,28]. The required aminoalkyne linker **6a** was then prepared in multi-gram quantities starting with selective propargylation of diethylene glycol **9a**. Subsequent tosylation of the remaining alcohol functionality [29] followed by treatment with NaN_3_ in the presence of TBAI afforded the corresponding azidoalkyne [30], which was reduced under Staudinger reduction conditions using solid phase triphenylphosphine to give **6a** (Scheme 2A) [24]. The linker **6a** was then reacted with sulfone **5** at 110 °C using microwave irradiation to afford **3a** (Scheme 2A).

#### 2.1.2. Synthesis of the *^t^*Bu-MTX-Azides **4**

*tert*-Butyl methotrexate **8** was prepared in gram quantities according to literature methods [23]. The aminoalkyne linker **7b** was synthesised in an analogous manner to aminoalkyne linker **6a**. Triethylene glycol **9b** was converted to the ditosylated analogue [29] and treated with NaN_3_ to afford the corresponding diazide [30] (Scheme 2B). Staudinger reduction of one of the diazide groups in the presence of 1 equivalent of PPh_3_ and 1 N HCl afforded a pure sample of **7b** following an acid-base work-up [31]. Aminoazide **7b** was then coupled to *^t^*Bu-MTX **8** to give **4b** (Scheme 2B).

#### 2.1.3. Assembly of CID **MTX-Cmpd2.2**

**Scheme 3:**
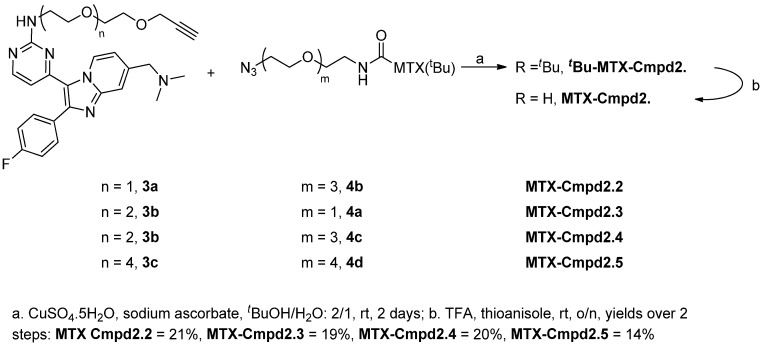
Hüisgen 1,3-dipolar cycloaddition and deprotection to **MTX-Cmpd2.2**.

With components **3a** and **4b** in hand, coupling *via* the Hüisgen 1,3-dipolar cycloaddition reaction (an example of a click reaction [32,33,34,35]) using copper(II) sulphate and sodium ascorbate was attempted (Scheme 3). Despite achieving the required transformation under these reaction conditions, problems were initially encountered in isolating the required *tert*-butyl-protected CID in a pure form, given its high molecular weight and polarity. After extensive optimisation of the purification procedure, ***^t^*Bu-MTX-Cmpd2.2** was purified using column chromatography on normal phase silica gel eluting with a mixture of DCM, MeOH and an aqueous NH_4_OH solution. Subsequent treatment of ***^t^*Bu-MTX-Cmpd2.2** with TFA in the presence of thioanisole provided **MTX-Cmpd2.2** (Scheme 3).

#### 2.1.4. Y3H results with CIDs **MTX-Cmpd2.1** and **MTX-Cmpd2.2**

**Figure 4:**
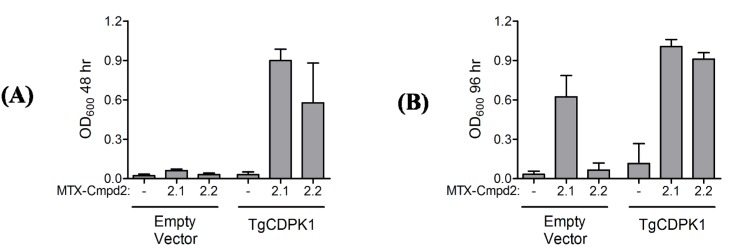
Comparison of the interaction of **MTX-Cmpd2.1** and **MTX-Cmpd2.2** with a known kinase target of compound **2**. *LEU2* reporter activation growth assay with yeast expressing a TgCDPK1-AD fusion protein in the presence of **MTX-Cmpd2.1** and **MTX-Cpmd2.2**. Growth was assessed at (**A**) 48 hour and (**B**) 96 hour by measuring the OD_600_ of the yeast culture. Samples treated with the DMSO vehicle were used as negative controls. Mean values ± SD are shown (n = 3).

The biological activity of **MTX-Cmpd2.1** and **MTX-Cmpd2.2** were assessed using our standard Y3H growth assays with yeast expressing *T. gondii* calcium-dependent protein kinase1 (TgCDPK1) fused to the activation domain. TgCDPK1 has previously been identified as a target of compound **2** [9,28]. Empty vector (the AD vector without TgCDPK1 and containing a stop codon immediately downstream of the multiple cloning site) was used as a negative control. Gratifyingly, both CIDs showed a robust Y3H interaction with TgCDPK1 based on *LEU2* reporter activation in 48 hour growth assays (Figure 4A), consistent with the view that the Y3H system can tolerate incorporation of a 1,2,3-triazole ring in the linker unit. Interestingly, at longer time points **MTX-Cmpd2.2** showed less background growth in the empty vector control than **MTX-Cmpd2.1** (Figure 4B), suggesting that the use of the triazole-containing CID **MTX-Cmpd2.2** may lead to a reduction in the number of false positive hits associated with a Y3H screen by decreasing the background growth of yeast expressing non-interacting targets.

Given the observation that incorporation of a triazole ring in the linker unit was tolerated and to test the modular nature of our approach, it was decided to try and further optimise the interaction between MTX and the DHFR-DBD fusion protein and compound **2** and the TgCDPK1-AD fusion protein by preparing CIDs with: (i) an alternative positioning of the triazole ring and (ii) a modified linker length. The synthesis of **MTX-Cmpd2.3-2.5** was achieved rapidly by mixing and matching the components **3** and **4** that contained varying numbers of PEG units (n and m respectively) that had been prepared separately in gram quantities (Scheme 3).

### 2.2. Synthesis and Analysis of Additional **MTX-Cmpd2** CIDs

**Figure 5:**
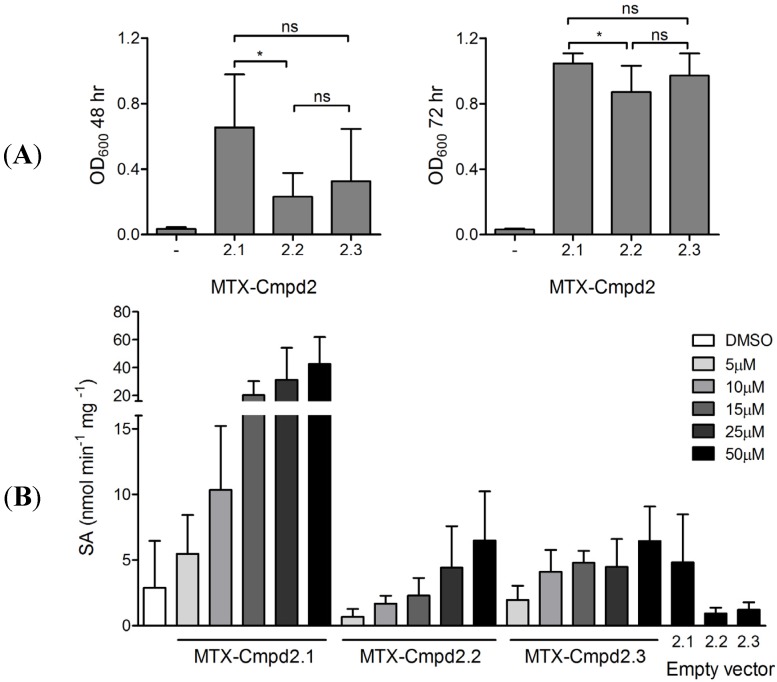
The effect of changing the triazole ring position in the CID linker. **(A**) Growth assay comparing the ability of **MTX-Cmpd2.2** and **MTX-Cmpd2.3** to turn on the *LEU2* reporter gene in the presence of the TgCDPK1-AD fusion protein as the target. Growth was assessed at 48 and 72 h by measuring the OD_600_ of the yeast culture. Samples treated with the DMSO vehicle were used as negative controls. Mean values ± SD are shown (n ≥ 5) and were compared using paired Student’s t-test. (**B**) β-Galactosidase assay comparing the ability of **MTX-Cmpd2.1**, **MTX-Cmpd2.2** and **MTX-Cmpd2.3** to turn on the *LacZ* reporter gene in the presence of TgCDPK1-AD as the target *^a^*.

To determine whether the positioning of the triazole ring affected the interaction of the CID with its target protein, **MTX-Cmpd2.3** was rapidly prepared by the reaction of **3b** and **4a** (Scheme 2 and Scheme 3). **MTX-Cmpd2.3** was then compared to **MTX-Cmpd2.2** and **MTX-Cmpd2.1** in the Y3H system by evaluating the activation of the reporter genes *LEU2* (growth assay; Figure 5A) and *LacZ* (β-galactosidase assay; Figure 5B). The two triazole-containing CIDs behaved similarly in both assays (compare **MTX-Cmpd2.2** and **MTX-Cmpd2.3** in Figure 5A and 5B), demonstrating that the position of the triazole ring has little, if any, effect on the interaction of the CID with the two fusion proteins. In the *LEU2* reporter assay, the triazole-containing CIDs tended to show less growth at 48 h than **MTX-Cmpd2.1**, but all three supported similar growth at 72 h (Figure 5A). In the more quantitative *LacZ* reporter assay, the triazole-containing CIDs showed less reporter activation than **MTX-Cmpd2.1**, but with similar dose-response curves (Figure 5B). The triazole-containing CIDs once again showed less non-specific reporter activation with the empty vector than **MTX-Cmpd2.1** (Figure 5B).

*^a^* Dose response curves of each CID are presented with the lowest dose corresponding to the vehicle (DMSO) treated samples. Mean values ± SD are shown (n ≥ 3). The differences in β-galactosidase activity between the DMSO-treated samples and the lowest concentrations of CID were not statistically significant. Note that in the 72h *LEU2*based growth assay (panel 5A), *i.e.*, the reporter and assay conditions most similar to those used for cDNA library screening, growth in the presence of DMSO is significantly different from growth in the presence of any of the CIDs tested.

**Figure 6:**
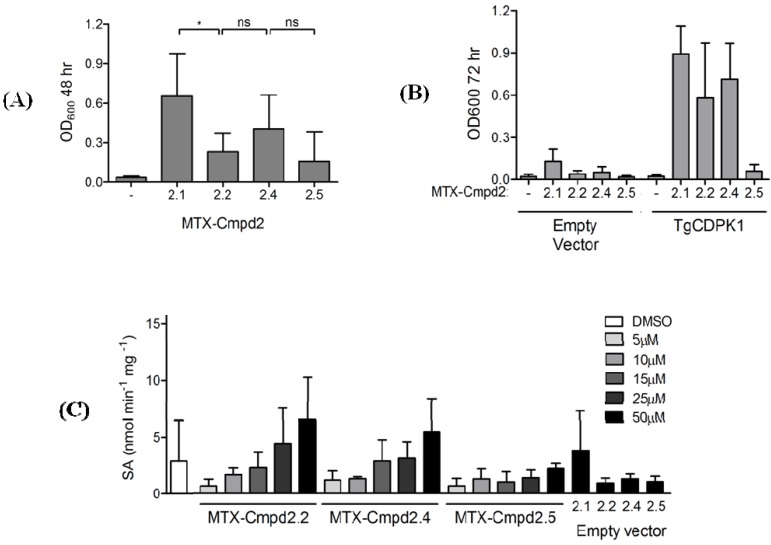
Effect of CID linker length on the Y3H interaction between compound **2**-based CIDs and TgCDPK1. (**A**) *LEU2* reporter activation growth assay with yeast expressing a TgCDPK1-AD fusion protein in the presence of the **MTX-Cmpd2** CID series with different linker lengths. Samples treated with the DMSO vehicle were used as negative controls. Mean values ± SD are shown (n ≥ 5) and compared using paired Student’s t-test; (**B**) *LEU2* reporter activation growth assay as in panel A using yeast expressing TgCDPK1-AD; (**C**) β-Galactosidase assay in the presence of the **MTX-Cmpd2** CID series with different linker lengths. Dose response curves with each CID are presented with the lowest dose corresponding to the vehicle (DMSO) treated samples. Mean values ± SD are shown (n ≥ 3).

With confirmation that changes in the positioning of the triazole-PEG linker unit were compatible with this Y3H assay, the influence of linker length was investigated. Two new CIDs, **MTX-Cmpd2.4** and **MTX-Cmpd2.5** were prepared from **3b** and **4c** and **3c** and **4d** respectively (Scheme 3). The intermediate length CID, **MTX-Cmpd2.4**, gave a positive Y3H interaction with the TgCDPK1-AD fusion protein in the growth assay, but the longest CID of the series, **MTX-Cmpd2.5,** failed to support a robust Y3H response; this was particularly evident after 72 h (Figure 6A,B). A similar result was observed in the *LacZ* reporter assay (Figure 6C).

**MTX-Cmpd2.4**, therefore, appears to be the optimal size for the ternary complex with TgCDPK1, as this CID consistently supported slightly (though not statistically significant) better growth than **MTX-Cmpd2.2** and **MTX-Cmpd2.5** (Figure 6A,B). CIDs with a linker unit longer than that present in **MTX-Cmpd2.4** are likely to be suboptimal because of the entropic cost that must be paid to correctly locate the two functional domains of the transcription factor. In an analogous way to the observations reported here for successful Y3H interactions, variations in a linker unit that maintains the two domains in a fusion protein have been shown to influence significantly the appropriate separation and folding of each domain [36]. In both cases it seems likely that the separation provided by the linker unit not only allows correct folding of the two domains in the fusion protein (or the DNA binding and activation domains in the Y3H system) but also affects the overall stability of the complex by changing its hydrophobicity profile [37]. Whilst small linkers restrict the conformational space of the individual domains, longer linkers may be more exposed to the solvent resulting in the inherent properties of the linker unit such as its hydrophobicity or secondary structure potentially coming into play. These could in turn affect operationally important parameters such as CID solubility, uptake or stability. It seems likely that the optimal linker length will change depending on the small molecule-protein pair being studied and therefore the ability to prepare families of CIDs relatively quickly will be important for the applications of Y3H (for example in the detailed study of specific interactions between a bioactive molecule under study and a target by defining important residues in a binding site) [38,39,40].

## 3. Experimental

### 3.1. General

Thin layer chromatography (TLC) analysis was performed using glass plates coated with silica gel (with fluorescent indicator UV_254_). Developed plates were air dried and analysed under a UV lamp (254/365 nm). Flash chromatography was performed using silica gel (40–63 µm, Fluorochem). Low resolution (LR) and high resolution (HR) electrospray mass spectral (ES-MS) analyses were acquired by electrospray ionisation (ESI), electron impact (EI) or chemical ionisation (CI). These were acquired within the School of Chemistry, University of St Andrews. Nuclear magnetic resonance (NMR) spectra were acquired at room temperature on either a Bruker Avance 300 (^1^H, 300.1 MHz; ^13^C, 75.5 MHz), a Bruker Avance II 400 (^1^H, 400.1 MHz; ^13^C, 100.6 MHz), a Bruker Avance 500 (^1^H, 500 MHz; ^13^C, 125.7 MHz) or a Bruker Avance III 500 (^1^H, 500.1 MHz, ^13^C, 125.7 MHz) spectrometer and in the deuterated solvent stated. All NMR spectra were acquired using the deuterated solvent as the lock. Coupling constants (*J*) are quoted in Hz and are recorded to the nearest 0.1 Hz. The following abbreviations are used; s, singlet; d, doublet; t, triplet; m, multiplet and br, broad. Chemical shifts are expressed as δ in units of ppm. ^13^C-NMR spectra were recorded under the same conditions and solvents using the PENDANT sequence mode. Data processing was carried out using the TOPSPIN 2 NMR program (Bruker UK Ltd).

### 3.2. Synthetic Procedures

#### 3.2.1. General Procedure A: Synthesis of Compound **2**-based alkyne **3**

Sulfone **5** [23] was added to a solution of aminoalkyne **6** (500 mg, 3 equiv.) in CH_3_CN (7 mL). The reaction mixture was then irradiated in the microwave for 1 hour at 100 °C (PSI ~ 50). The reaction mixture was then concentrated in *vacuo* to give an oil which was purified by column chromatography (DCM/MeOH: 98/2 to 95/5).

*Compound* **2***-based alkyne* **3a**. General Procedure A was followed using aminoalkyne **6a** (500 mg, 3.49 mmol) to give **3a** as an amber oil (370 mg, 0.72 mmol, 62%). ^1^H-NMR (400 MHz, CDCl_3_) δ 2.32 (s, 6H), 2.46 (t, *J* = 2.3 Hz, 1H), 3.46–3.80 (m, 10H), 4.22 (d, *J* = 2.3 Hz, 2H), 5.62 (br. s, 1H), 6.43 (d, *J* = 5.3 Hz, 1H), 7.00–7.16 (m, 3H), 7.55 (s, 1H), 7.60–7.68 (m, 2H), 8.11 (d, *J* = 5.3 Hz, 1H), 9.45 (d, *J* = 7.3 Hz, 1H). ^13^C-NMR (126 MHz, CDCl_3_) δ 45.3, 58.5, 63.4, 69.0, 69.1, 70.3, 74.7, 79.6, 109.2, 114.3, 115.7, 116.0, 118.6, 126.9, 131.2, 131.2, 138.6, 146.4, 147.7, 157.2, 157.9, 161.8, 162.4, 164.2. HRMS (ESI) calculated for C_27_H_29_FN_6_O_2_Na: 511.2234; found 511.2241.

*Compound* **2***-based alkyne* **3b**. General Procedure A was followed using aminoalkyne **6b** (500 mg, 2.67 mmol) to give **3b** as an amber oil (295 mg, 0.55 mmol, 62%). ^1^H-NMR (400 MHz, CDCl_3_) δ 2.34 (s, 6H), 2.44–246 (m, 1H), 3.60 –3.77 (m, 14H), 4.21 (d, *J* = 2.4 Hz, 2H), 5.65 (m, 1H), 6.43 (d, *J* = 5.3 Hz, 1H), 7.11 (t, *J* = 8.7 Hz, 2H), 7.26 (d, *J* = 1.0 Hz, 1H), 7.54 (s, 1H), 7.64 (dd, *J* = 8.9, 5.5 Hz, 2H), 8.10 (d, *J* = 5.3 Hz, 1H), 9.44 (d, *J* = 7.3 Hz, 1H). ^13^C-NMR (126 MHz, CDCl_3_) δ 45.3, 58.4, 63.4, 69.1, 69.8, 70.1, 70.5, 70.6, 74.6, 77.0, 109.8, 114.3, 115.5, 115.7, 117.9, 126.9, 131.2, 131.2, 138.5, 146.4, 147.6, 157.4, 157.9, 161.8, 162.1, 164.3. HRMS (ESI) calculated for C_29_H_33_FN_6_O_3_Na: 555.2496; found 555.2499.

*Compound* **2***-based alkyne* **3c**. General Procedure A was followed using aminoalkyne **6c** (500 mg, 1.81 mmol) to give **3c** as an amber oil (225 mg, 0.362 mmol, 60%). ^1^H-NMR (300 MHz, CDCl_3_) δ 2.23 (s, 6H), 2.44–2.46 (m, 1H), 3.48–3.94 (m, 22H), 4.18–4.20 (m, 2H), 5.75 (t, *J* = 5.0 Hz, 1H), 6.37 (d, *J* = 5.3 Hz, 1H), 6.93–7.18 (m, 3H), 7.49–7.69 (m, 3H), 8.11 (d, *J* = 5.3 Hz, 1H), 9.45 (d, *J* = 7.2, Hz, 1H). ^13^C-NMR (101 MHz, CDCl_3_) δ 45.3, 58.4, 63.4, 69.1, 69.4, 70.1, 70.3, 70.4, 70.4, 70.5, 70.5, 70.6, 74.5, 79.6, 109.8, 114.2, 115.6, 116.3, 118.0, 126.9, 131.2, 131.2, 138.6, 146.4, 147.7, 157.4, 157.9, 161.8, 162.1, 164.2. HRMS (ESI) calculated for C_33_H_41_FN_6_O_5_Na: 643.3020, found 643.2970.

#### 3.2.2. General Procedure B: Synthesis of *^t^*Bu-MTX-azide Components **4**

Dry diisopropylethylamine (2 equiv.) was added to a suspension of methotrexate (MTX) **8** [23] in anhydrous DMF under a nitrogen atmosphere. TPTU was then added to the flask and the resulting mixture left to stir at room temperature overnight. In a separate flask, diisopropylethylamine (2 equiv.) was added to a suspension of the aminoazide **7** (1.1 equiv.) in anhydrous DMF (12 mL) under a nitrogen atmosphere. The activated acid solution was then added to the flask *via* cannula and the flask rinsed with anhydrous DMF (10 mL). The resulting mixture was then stirred at room temperature overnight before being concentrated in *vacuo* to give **4** as a dark brown oil. Purification was achieved *via* column chromatography (DCM/MeOH: 98/2 to 9/1).

*^t^Bu-MTX-Azide* **4a**. General Procedure B was followed using aminoazide **7a** (2.0 g, 0.015 mol) to give **4a** (5.17 g, 8.31 mmol, 61%) as a dark orange solid. ^1^H-NMR (500 MHz, DMSO) δ 1.40 (s, 9H), 1.90 (m, 1H), 1.92–2.01 (m, 1H), 2.13–2.26 (m, 2H), 3.12–3.25 (m, 5H), 3.39–3.67 (m, 6H), 4.18–4.22 (m, 1H), 4.80 (s, 2H), 6.73–6.80 (br. s, 2H), 6.82–6.88 (m, 2H), 7.70–7.73 (m, 2H), 7.87–7.99 (m, 1H), 8.27 (d, *J* = 7.3 Hz, 1H), 8.59 (s, 1H). ^13^C-NMR (126 MHz, DMSO) δ 26.9, 28.1, 32.3, 38.9, 50.3, 53.4, 55.3, 69.3, 69.4, 70.2, 80.7, 111.5, 121.6, 122.0, 129.3, 147.3, 149.5, 151.3, 154.8, 162.2, 163.1, 166.7, 172.0, 172.1. HRMS (ESI) calculated for C_28_H_38_N_12_O_5_Na: 645.2986; found 645.2997.

*^t^Bu-MTX-Azide* **4b**. General Procedure B was followed using aminoazide **7b** (2.1 g, 0.012 mol) to give **4b** as an orange solid (3.9 g, 5.85 mmol, 56%). ^1^H-NMR (300 MHz, DMSO) δ 1.39 (s, 9H), 1.82–2.07 (m, 2H), 2.14–2.26 (m, 2H), 3.12–3.25 (m, 5H), 3.39–3.66 (m, 10H), 4.14–4.28 (m, 1H), 4.80 (s, 2H), 6.75–6.87 (m, 2H), 6.96 (br. s, 2H), 7.68–7.78 (m, 2H), 7.86–8.07 (m, 1H), 8.27 (d, *J* = 7.3 Hz, 1H), 8.60 (s, 1H). ^13^C-NMR (126 MHz, DMSO) δ 26.9, 28.1, 32.3, 39.0, 39.6, 50.4, 53.4, 55.3, 69.6, 69.7, 70.0, 80.7, 111.5, 121.6, 121.9, 129.4, 147.1, 149.5, 151.3, 162.5, 163.2, 166.8, 172.0, 172.1. HRMS (ESI) calculated for C_30_H_42_N_12_O_6_Na: 689.3248; found 689.3262.

*^t^Bu-MTX-Azide* **4c**. General Procedure B was followed using aminoazide **7c** (2.0 g, 9.16 mmol) to give **4c** as a bright yellow solid (2.9 g, 4.16 mmol, 50%). ^1^H-NMR (300 MHz, DMSO) δ 1.39 (s, 9H), 1.81–2.12 (m, 2H), 2.18–2.22 (m, 2H), 3.11–3.24 (m, 5H), 3.34–3.66 (m, 14H), 4.18–4.21 (m, 1H), 4.78 (s, 2H), 6.61 (s, 2H), 6.77–6.86 (m, 2H), 7.62–7.77 (m, 2H), 7.90 (t, *J* = 5.5 Hz, 1H), 8.25 (d, *J* = 7.3 Hz, 1H), 8.56 (s, 1H). ^13^C-NMR (126 MHz, DMSO) δ 26.9, 28.1, 32.3, 39.0, 39.4, 50.4, 53.4, 55.3, 69.5, 69.7, 70.0, 70.1, 70.2, 70.2, 80.7, 111.5, 121.6, 122.1, 129.4, 147.9, 149.5, 151.3, 153.0, 161.4, 163.1, 166.7, 172.0, 172.1. HRMS (ESI) calculated for C_32_H_46_N_12_O_7_Na: 733.3510; found 733.3515.

*^t^Bu-MTX-Azide* **4d**. General Procedure B was followed using aminoazide **7d** (2.1 g, 8.00 mmol) to give **4d** as an orange solid (3.2 g, 4.24 mmol, 53%). ^1^H-NMR (500 MHz, DMSO) δ 1.39 (s, 9H), 1.80–2.07 (m, 2H), 2.15–2.24 (m, 2H), 3.10–3.19 (m, 5H), 3.32–3.69 (m, 18H), 4.18–4.21 (m, 1H), 4.78 (s, 2H), 6.65 (br. s, 2H), 6.81 (d, *J* = 8.6 Hz, 2H), 7.48 (br. s, 2H), 7.72 (d, *J* = 8.7 Hz, 2H), 7.88–7.94 (m, 1H), 8.25 (d, *J* = 7.3 Hz, 1H), 8.56 (s, 1H). ^13^C-NMR (126 MHz, DMSO) δ 26.4, 27.6, 31.7, 38.5, 49.9, 52.9, 54.8, 69.0, 69.2, 69.5, 69.6, 69.7, 69.7, 69.7, 80.2, 110.9, 121.0, 121.4, 128.8, 146.0, 149.1, 150.8, 155.0, 162.6, 162.7, 166.2, 171.5, 171.5. HRMS (ESI) calculated for C_34_H_50_N_12_O_8_Na: 777.3772; found 777.3767.

#### 3.2.3. General Procedure C: Coupling of **3** and **4**

Compound **2** based alkyne **3** (1.1 equiv.) was dissolved in a mixture of *^t^*BuOH/H_2_O (1/2). The *^t^*Bu-MTX-azide **4** was then added to the flask and the resulting mixture left to stir vigorously at room temperature until complete dissolution of both starting materials had occurred (*ca.* 30 min). Copper (II) sulphate pentahydrate (1.4 equiv.) was then added to the flask followed by sodium ascorbate (2.8 equiv.). The reaction mixture was then left to stir at room temperature for 24 h. At that time, an additional 1.4 equiv. of copper (II) sulphate pentahydrate were added to the flask followed once again by sodium ascorbate (2.8 equiv.). The reaction mixture was left to stir at room temperature for an additional 24 h. The reaction mixture was then concentrated in *vacuo* to give a dark solid. ***^t^*Bu-MTX-Cpmd2.2**–**2.5** was successfully isolated following column chromatography (DMC/MeOH/aq. NH_4_OH: 100/1/6 drops to 9/1/6 drops).

***^t^*Bu-MTX-Cpmd2.2**. General Procedure C was followed using **3a** (187 mg, 0.38 mmol, 1.1 equiv.) and **4b** (232 mg, 0.35 mmol) to give ***^t^*Bu-MTX-Cpmd2.2** as a film (100 mg, 0.087 mmol, 25%). ^1^H-NMR (500 MHz, DMSO) δ 1.38 (s, 9H), 1.86–1.88 (m, 1H), 1.98–2.03 (m, 1H), 2.19–2.27 (m, 2H), 2.98 (s, 3H), 3.15–3.80 (m, 29H), 4.15–4.25 (m, 1H), 4.48–4.63 (m, 4H), 4.80 (s, 2H), 6.30–6.40 (m, 1H), 6.81 (d, *J* = 6.7 Hz, 2H), 6.88–6.91 (m, 1H), 7.11–7.20 (m, 1H), 7.30 (t, *J* = 8.7 Hz, 2H), 7.62–7.78 (m, 4H), 7.81 (s, 1H). 7.90–7.92 (m, 1H), 8.03 (s, 1H), 8.18 (d, *J* = 5.2 Hz, 1H), 8.26 (d, *J* = 7.2 Hz, 1H), 8.59 (s, 1H). ^13^C-NMR (126 MHz, DMSO) δ 26.4, 27.6, 31.7, 38.4, 39.5, 39.9, 39.9, 40.0, 40.5, 42.7, 49.2, 52.9, 54.8, 63.4, 68.6, 68.9, 69.0, 69.3, 69.4, 69.6, 80.2, 109,1, 110.9, 114.7, 115.4, 115.6, 119.0, 121.1, 121.6, 124.2, 128.8, 131.1, 131.7, 143.7, 147.3, 147.4, 148.6, 149.0, 150.8, 159.0, 161.3, 161.4, 162.6, 163.6, 166.2, 171.5, 171.5, 171.9. HRMS (ESI) calculated for C_57_H_71_FN_18_O_8_Na: 1177.5584; found 1177.5598.

***^t^*Bu-MTX-Cpmd2.3**. General Procedure C was followed using **3b** (195 mg, 0.37 mmol, 1.1 equiv.) and **4a** (207 mg, 0.33 mmol) to give ***^t^*Bu-MTX-Cpmd2.3** as a film (97 mg, 0.084 mmol, 25%). ^1^H-NMR (300 MHz, DMSO) δ 1.37 (s, 9H), 1.80–1.88 (m, 1H), 1.90–1.97 (m, 1H), 2.15–2.20 (m, 2H), 3.06–3.23 (m, 6H), 3.36–3.65 (m, 25H), 4.18–4.21 (m, 1H), 4.46–4.50 (m, 4H), 4.77 (s, 2H), 6.29–6.32 (m, 1H), 6.67 (br. s, 2H), 6.80 (m, 2H), 7.05 (d, *J* = 7.1 Hz, 1H), 7.28 (t, *J* = 8.8 Hz, 2H), 7.58–7.75 (m, 5H), 7.86 (t, *J* = 5.7 Hz, 1H), 8.02 (s, 1H), 8.16–8.18 (m, 1H), 8.24–8.26 (m, 1H), 8.55 (s, 1H). ^13^C-NMR (126 MHz, DMSO) δ 26.4, 27.6, 31.7, 38.4, 38.4, 39.4, 39.9, 39.9, 40.0, 43.1, 49.2, 52.9, 54.8, 63.4, 68.6, 68.8, 69.2, 69.5, 69.6, 80.2, 111.1, 114.7, 115.4, 115.6, 119.0, 121.1, 121.5, 124.2, 128.8, 131.1, 131.6, 143.7, 147.9, 148.6, 149.1, 150.8, 159.1, 161.1, 162.1, 162.7, 163.6, 166.2, 171.5, 171.5, 171.9. HRMS (ESI) calculated for C_57_H_71_FN_18_O_8_Na: 1177.5584; found 1177.5742.

***^t^*Bu-MTX-Cpmd2.4**. General Procedure C was followed using **3b** (220 mg, 0.41 mmol, 1.1 equiv.) and **4c** (267 mg, 0.37 mmol) to give ***^t^*Bu-MTX-Cpmd2.4** as a film (130 mg, 0.104 mmol, 28%). ^1^H-NMR (500 MHz, DMSO) δ 1.38 (s, 9H), 1.82–1.89 (m, 1H), 1.99–2.02 (m, 1H), 2.19 (m, 2H), 2.10–3.19 (m, 5H), 3.33–3.61 (m, 32H), 3.78 (dt, *J* = 9.6, 5.6 Hz, 2H), 4.19–4.21 (m, 1H), 4.49–4.51 (m, 4H), 4.79 (s, 2H), 6.30–6.34 (m, 1H), 6.59–6.62 (m, 1H), 6.78–6.87 (m, 2H), 7.11 (d, *J* = 7.5 Hz, 1H), 7.30 (t, *J* = 8.6 Hz, 2H), 7.52 (s, 1H), 7.69–7.81 (m, 4H), 7.91 (t, *J* = 5.8 Hz, 1H), 8.04 (s, 1H), 8.18 (d, *J* = 5.2 Hz, 1H), 8.23–8.29 (m, 1H), 8.58 (s, 1H). ^13^C-NMR (126 MHz, DMSO) δ 26.4, 27.6, 31.7, 38.5, 39.5, 39.9, 40.0, 40.1, 43.0, 49.2, 52.9, 54.8, 59.7, 63.4, 68.6, 68.9, 69.0, 69.5, 69.5, 69.6, 69.6, 69.7, 80.2, 111.0, 114.7, 115.4, 115.6, 118.9, 119.0, 121.1, 121.5, 124.2, 128.8, 131.1, 131.7, 143.7, 146.3, 148.6, 149.0, 150.8, 159.0, 161.4, 162.6, 163.6, 166.2, 171.5, 171.5, 171.9. HRMS (ESI) calculated C_61_H_80_FN_18_O_10_ 1243.6289 found 1243.6310. 

***^t^*Bu-MTX-Cpmd2.5**. General Procedure C was followed using **3d** (217 mg, 0.35 mmol, 1.1 equiv.) and **4d** (220 mg, 0.29 mmol) to give ***^t^*Bu-MTX-Cpmd2.5** as a film (92 mg, 0.067 mmol, 21%). ^1^H-NMR (500 MHz, DMSO) δ 1.38 (s, 9H), 1.83–2.03 (m, 2H), 2.18–2.21 (m, 2H), 3.12–3.23 (m, 5H), 3.29–3.61 (m, 42H), 3.78–3.81 (m, 2H), 4.16–4.27 (m, 1H), 4.48–4.51 (m, 4H), 4.77 (s, 2H), 6.32–6.35 (m, 1H), 6.66 (br. s, 2H), 6.80–6.82 (m, 2H), 7.03–7.05 (m, 1H), 7.28 (t, *J* = 8.8 Hz, 2H), 7.43 (s, 1H), 7.56 (s, 1H), 7.61–7.74 (m, 4H), 7.89 (t, *J* = 5.7 Hz, 1H), 8.03 (s, 1H), 8.15 (d, *J* = 5.2 Hz, 1H), 8.24 (d, *J* = 7.2 Hz, 1H), 8.55 (s, 1H). ^13^C-NMR (126 MHz, DMSO) δ 26.4, 27.6, 31.7, 38.5, 39.4, 39.9, 39.9, 40.0, 40.5, 43.7, 49.2, 52.9, 54.8, 57.4, 60.1, 60.3, 63.4, 66.3, 68.4, 68.6, 68.9, 69.0, 69.4, 69.5, 69.5, 69.6, 69.7, 72.3, 77.1, 80.2, 111.0, 114.7, 115.4, 115.6, 119.0, 121.0, 121.5, 124.2, 128.8, 131.1, 131.6, 143.7, 146.2, 148.1, 149.1, 150.8, 162.6, 166.2, 171.5, 171.5. HRMS (ESI) calculated C_67_H_91_FN_18_O_13_Na 1397.6895 found 1397.6901.

#### 3.2.4. General Procedure D: Boc Deprotection of ***^t^*Bu-MTX-Cmpd2**

Thioanisole (30 µL) was added to a solution of ***^t^*Bu-MTX-Cmpd2** followed by TFA (30 µL). The resulting mixture was then stirred at room temperature overnight before being concentrated in *vacuo* to give a brown solid. This was then suspended in cyclohexane to remove any traces of thioanisole and the resulting mixture concentrated in *vacuo* to give a yellow/pale orange film.

**MTX-Cpmd2.2**. General Procedure D was followed using ***^t^*Bu-MTX-Cpmd2.2** (90 mg, 0.090 mmol) to give **MTX-Cpmd2.2** as a yellow amorphous solid (84 mg, 0.076 mmol, 85%). ^1^H-NMR (500 MHz, DMSO) δ 1.89–2.10 (m, 2H), 2.14–2.23 (m, 2H), 2.82 (s, 6H), 3.05–3.62 (m, 19H), 3.71–3.82 (m, 2H), 4.28–4.32 (m, 1H), 4.42–4.52 (m, 6H), 4.87 (s, 2H), 6.32–6.42 (m, 1H), 6.81 (d, *J* = 8.3 Hz, 2H), 7.06 (s, 1H), 7.14–7.36 (m, 4H), 7.60–7.76 (m, 4H), 7.91 (d, *J* = 6.2 Hz, 1H), 8.04–8.08 (m, 1H), 8.17–8.21 (m, 1H), 8.31 (d, *J* = 7.4 Hz, 1H), 8.71 (s, 1H), 9.08 (s, 1H), 9.29 (s, 1H). ^13^C-NMR (126 MHz, DMSO) δ 26.5, 31.9, 38.4, 39.8, 40.6, 49.2, 52.2, 54.7, 58.4, 60.7, 63.4, 68.6, 69.3, 69.4, 69.6, 69.7, 70.4, 72.3, 108.8, 111.1, 115.6, 115.7, 116.1, 119.4, 120.1, 121.7, 122.1, 124.2, 124.7, 125.8, 129.2, 129.9, 130.6, 131.3, 138.7, 143.7, 144.2, 145.2, 145.6, 148.7, 149.2, 150.6, 151.1, 155.8, 157.3, 161.4, 163.7, 166.1, 171.7, 173.8. HRMS (ESI) calculated for C_53_H_64_FN_18_O_8_: 1099.5139; found 1099.5127.

**MTX-Cpmd2.3**. General Procedure D was followed using ***^t^*Bu-MTX-Cpmd2.3** (35 mg, 0.030 mmol) to give **MTX-Cpmd2.3** as a dark orange amorphous solid (24 mg, 0.023 mmol, 76%). ^1^H-NMR (400 MHz, DMSO) δ 1.85–2.06 (m, 2H), 2.18–2.22 (m, 2H), 2.85 (s, 6H), 3.20–3.79 (m, 21H), 4.33–4.42 (m, 1H), 4.43–4.55 (m, 6H), 4.90 (s, 2H), 6.31–6.41 (m, 1H), 6.86 (d, *J* = 9.1 Hz, 2H), 7.11–7.22 (m, 1H), 7.24–7.39 (m, 4H), 7.66–7.81 (m, 4H), 7.88–7.95 (m, 1H), 8.06 (d, *J* = 7.2 Hz, 1H), 8.24 (d, *J* = 5.3 Hz, 1H), 8.34 (d, *J* = 7.2 Hz, 1H), 8.74 (s, 1H), 9.10 (s, 1H), 9.30 (s, 1H). ^13^C-NMR (126 MHz, DMSO) δ 26.5, 31.9, 38.4, 39.4, 40.1, 49.7, 52.7, 55.3, 58.6, 60.6, 63.9, 68.5, 69.4, 69.6, 69.8, 70.1, 108.8, 111.6, 115.5, 115.6, 116.10, 119.7, 120.5, 121.7, 122.2, 124.7, 125.2, 129.4, 130.9, 131.2, 138.5, 143.7, 144.2, 145.3, 148.7, 149.1, 150.6, 151.2, 155.8, 157.3, 161.3, 162.9, 163.1, 166.1, 166.6, 171.8, 173.9. HRMS (ESI) calculated for C_53_H_63_FN_18_O_8_Na: 1121.4958; found 1121.4946.

**MTX-Cpmd2.4**. General Procedure D was followed using ***^t^*Bu-MTX-Cpmd2.4** (90 mg, 0.070 mmol) to give **MTX-Cpmd2.4** as a yellow/orange amorphous solid (56 mg, 0.045 mmol, 65%) ^1^H-NMR (500 MHz, DMSO) δ 1.89–2.10 (m, 2H), 2.18–2.25 (m, 2H), 3.16–3.66 (m, 37H), 4.25–4.32 (m, 1H), 4.36–4.58 (m, 6H), 4.87 (s, 2H), 6.31–6.42 (m, 1H), 6.76–6.89 (m, 2H), 7.07–7.41 (m, 5H), 7.60–7.84 (m, 4H), 7.90–7.92 (m, 1H), 8.03 (d, *J* = 10.5 Hz, 1H), 8.20 (d, *J* = 5.5 Hz, 1H), 8.30 (d, *J* = 7.3 Hz, 1H), 8.71 (s, 1H), 9.07 (s, 1H), 9.28 (s, 1H). ^13^C-NMR (126 MHz, DMSO) δ 26.5, 31.9, 38.5, 39.0, 39.2, 40.1, 42.0, 49.2, 52.2, 54.8, 58.9, 63.4, 68.6, 68.9, 69.0, 69.5, 69.5, 69.6, 69.6, 69.7, 108.8, 111.1, 115.5, 115.7, 120.0, 121.4, 121.6, 122.6, 124.2, 124.7, 125.9, 126.5, 128.9, 129.5, 131.2, 138.0, 138.6, 143.7, 145.9, 148.7, 149.1, 150.6, 151.1, 161.4, 162.6, 166.1, 171.7, 173.7. HRMS (ESI) calculated for C_57_H_72_FN_18_O_10_: 1187.5663; found 1187.5654.

**MTX-Cpmd2.5**. General Procedure D was followed using ***^t^*Bu-MTX-Cpmd2.5** (35 mg, 0.030 mmol) to give **MTX-Cpmd2.5** as an dark orange amorphous solid (28 mg, 0.021 mmol, 69%). ^1^H-NMR (300 MHz, DMSO) δ 1.82–2.26 (m, 6H), 2.82 (s, 6H), 3.21–3.85 (m, 41H), 4.18–4.21 (m, 1H), 4.33–4.59 (m, 6H), 4.88 (s, 2H), 6.32–6.42 (m, 1H), 6.82 (d, *J* = 8.6 Hz, 2H), 7.07–7.37 (m, 5H), 7.59–7.79 (m, 4H), 7.88–7.92 (m, 1H), 8.04 (d, *J* = 3.0 Hz, 1H), 8.18–8.21 (m, 1H), 8.31 (d, *J* = 7.2 Hz, 1H), 8.71 (s, 1H), 9.10 (s, 1H), 9.29 (s, 1H). ^13^C-NMR (126 MHz, DMSO) δ 27.0, 32.4, 38.99, 39.1, 39.4, 40.2, 42.4, 49.7, 52.7, 55.2, 57.7, 60.6, 63.9, 68.9, 69.1, 69.4, 69.5, 69.9, 70.0, 70.1, 70.2, 70.3, 72.8, 80.8 102.0, 108.8, 109.7, 111.6, 115.8, 116.0, 120.2, 121.8, 122.4, 124.3, 124.7, 125.8, 129.4, 131.5, 138.2, 144.2, 146.2, 148.1, 149.2, 151.1, 151.6, 156.4, 161.1, 163.1, 166.6, 172.2, 174.2. HRMS (ESI) calculated C_63_H_83_FN_18_O_13_Na 1341.6269 found 1341.6091.

### 3.3. Reporter Activation Assays

Growth assays to test the activation of the *LEU2* reporter in the yeast strain V784Y were done in liquid culture as described previously [23]. Briefly, assays were performed by diluting the yeast from a saturated overnight culture to 2 × 10^5^ cells/well in a sterile flat-bottom clear 96-well plate (Becton-Dickinson, Franklin Lakes, NJ, USA) with 150 µL of the appropriate selective media containing either CID (10 µM unless otherwise indicated) or the equivalent amount of DMSO (1% v/v). The plates were wrapped with parafilm and incubated at 30 °C with shaking at 80 rpm. Every 24 h the absorbance at 600 nm was measured using a pre-heated plate reader (BioTek, Winooski, VT, USA). Cell suspensions were mixed by pipetting prior to each measurement.

To measure activation of the *LacZ* gene, the standard liquid β-Galactosidase assay described in the Yeast Protocols Handbook (PT3024-1 Clontech, Mountain View, CA, USA) was adapted to 150 µL cultures grown in 96-well plates. Briefly, saturated overnight cultures were diluted 1:100 in SC +Gal/Raff -his-trp-ura media in sterile flat-bottom 96-well plates and incubated at 30 °C for 72 h. Yeast cultures were collected and centrifuged at 21 °C at 16,000 × *g* for 5 min. The pellets were washed once with double-distilled water and resuspended in freshly prepared breaking buffer containing 1x protease inhibitor mix (P8340 Sigma, Gillingham, Dorset, UK), 100 mM Tris-HCl pH8.0, 1 mM DTT and 20% v/v glycerol. Acid-washed beads were added to each tube to fill the volume up to the meniscus and the cells were broken by 10 × 15 sec pulses of vortexing, each pulse followed by 1 min incubation on ice. The spheroblast suspension was then solubilized in 0.1% w/v SDS for 10 min at 21 °C. 25 µL of each lysate was used to measure total protein concentration by the standard microtiter Bradford assay (BioRad, Hercules, CA, USA). The rest of the extract was used for the β-galactosidase assay using CPRG (Roche, Indianapolis IN, USA) as substrate, as described in the Yeast Protocols Handbook (Clontech PT3024-1).

## 4. Conclusions

The development of a modular approach to the synthesis of triazole-containing chemical inducers of dimerisation (CIDs) has been described and used to prepare four CIDs, **MTX-Cmpd2.2**–**2.5**. The ability to prepare in a rapid manner a small family of CIDs enables the researcher to explore which linker length is the most suitable for a particular target identification study. In this case, our compound **2**-based CIDs were then compared using the Y3H approach with three of them giving a strong positive interaction with a known target of compound **2**, TgCDPK1. Interestingly at longer time points, the triazole-containing CIDs **MTX-Cmpd2.2-2.5**, all showed less background growth compared to PEG-containing CID, **MTX-Cmpd2.1**. This novel observation may have implications for future Y3H screening campaigns. The modular nature of the synthetic strategy used here will help to overcome the CID synthesis challenges currently encountered and should contribute to the Y3H approach reaching its full potential as an unbiased target identification strategy.

## Supplementary Materials

Detailed synthesis of aminoalkynes **6** and aminoazides **7** can be found in the supplementary information provided alongside ^1^H, ^13^C and 2D NMR spectra of ***^t^*Bu-MTX-cmpd2.2** and **MTX-Cmpd2.2**. This material is available free of charge at: http://www.mdpi.com/1420-3049/18/9/11639/s1.
