# Supplementary material for: A Modular Approach to Triazole-Containing Chemical Inducers of Dimerisation for Yeast Three-Hybrid Screening
Source: Molecules. 2013 Sep 23;18(9):11639–57. doi: 10.3390/molecules180911639 (PMC4031444; doi:10.3390/molecules180911639)
Supplement: Supplementary file 1 [file molecules-18-11639-s001.pdf]

Supplementary Materials

Synthesis of aminoalkynes **6a**–**d** (Scheme S1)

Scheme S1. Synthesis of aminoalkynes **6a**–**d**.

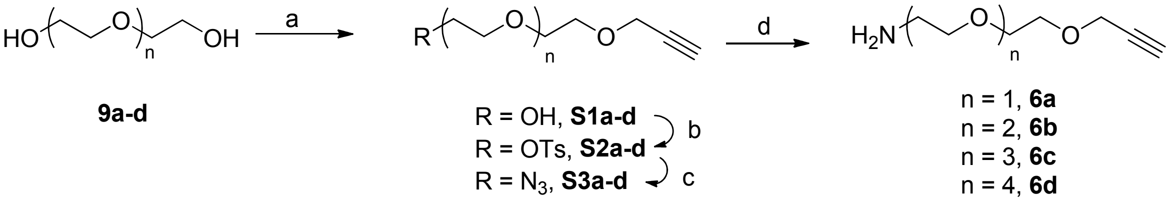

a. i. NaH, DMF, 0 °C; ii. propargyl bromide, rt, o/n; b. TsCl, KOH, DMF, 0 °C to rt, o/n; c. NaN_3, DMF, TBAI, 120 °C, o/n; d. i) PS-PPh_3, THF, o/n; ii) H_2O, rt, o/n, yields over 4 steps: **6a** = 27%, **6b** = 31%, **6c** = 28%, **6d** = 46%

General Procedure A: Propargylation of polyethylene glycol

Sodium hydride (1.5 eq.) was added under a nitrogen atmosphere to a cooled (0 °C) solution of polyethylene glycol **9** (1 eq.) in anhydrous DMF followed by a solution of propargyl bromide in toluene (80% wt, 3 eq.). The reaction mixture was stirred at room temperature for 2 days. It was then concentrated in *vacuo* to give an oil, which was purified by column chromatography (hexane/EtOAc).

2-(2-(Prop-2-yn-1-yloxy)ethoxy)ethanol **S1a** General procedure A was followed using diethylene glycol **9a** (3.0 mL, 0.030 mol) to yield **S1a** as a light amber oil (3.0 g, 0.019 mol, 65%). ^{1}H-NMR (300 MHz, $CDCl_3$) δ 2.38–2.51 (m, 1H), 3.55–3.81 (m, 10H), 4.20–4.22 (m, 2H). ^{1}H-NMR data were in accordance with the literature [1].

2-(2-(2-(Prop-2-yn-1-yloxy)ethoxy)ethoxy)ethanol **S1b**. General procedure A was followed using triethylene glycol **9b** (5.0 mL, 0.040 mol) to yield **S1b** (4.1 g, 0.023 mol, 58%) as a light amber oil. ^{1}H-NMR (300 MHz, $CDCl_3$) δ 2.40 - 2.44 (m, 1H), 3.56–3.79 (m, 12H), 4.21 (t, J = 2.2 Hz, 2H). ^{1}H-NMR data were in accordance with the literature [2].

3,6,9,12-Tetraoxapentadec-14-yn-1-ol **S1c**. General procedure A was followed using tetraethylene glycol **9c** (2.0 mL, 0.010 mol) to yield **S1c** as a light amber oil (1.4 g, 5.12 mmol, 51%). ^{1}H-NMR (300 MHz, $CDCl_3$) δ 2.43 (t, J = 2.4 Hz, 1H), 3.56–3.77 (m, 16H), 4.21 (d, J = 2.3 Hz, 2H). ^{1}H-NMR data were in accordance with the literature [3].

3,6,9,12,15-Tetraoxaoctadec-14-yn-1-ol **S1d**. General procedure A was followed using pentaethylene glycol **9d** (3.0 mL, 0.010 mol) to yield **S1d** as a yellow oil (2.4 g, 6.12 mmol, 61%). ^{1}H-NMR (400 MHz, $CDCl_3$) δ 2.42 (t, J = 2.4 Hz, 1H), 3.56–3.78 (m, 20H), 4.20 (d, J = 2.4 Hz, 2H). ^{1}H-NMR data were in accordance with the literature. ^{1}H-NMR data were in accordance with the literature [4].

General Procedure B: Tosylation of compounds **S1a**–**d**

Freshly ground potassium hydroxide (2 eq.) was added to a cooled (0 °C) solution of **S1** in anhydrous DCM under a nitrogen atmosphere. The mixture was then stirred at this temperature for an

hour before the slow addition of *p*-toluene sulfonylchloride (1.1 eq.). The reaction mixture was then stirred at room temperature overnight before being concentrated in *vacuo* to give a beige oily solid. The crude product was then taken up in EtOAc (25 mL) and the resulting suspension stirred at room temperature for 30 minutes. The insoluble solid was removed by filtration and the filtrate concentrated in *vacuo* to afford **S2**.

2-(2-(2-(Prop-2-yn-1-yloxy)ethoxy)ethoxy)ethanol **S2a**. General procedure B was followed using 2-(2-(prop-2-yn-1-yloxy)ethoxy)ethanol **S1a** (2.8 g, 0.02 mol) to give **S2a** as an amber oil (4.5 g, 0.018 mol, 89%). ^{1}H-NMR (300 MHz, $CDCl_3$) δ 2.42–2-45 (m, 4H), 3.55–3.76 (m, 6H), 4.10–4.25 (m, 4H), 7.29–7.39 (m, 2H), 7.79–7.81 (m, 2H). ^{1}H-NMR data were in accordance with the literature [1].

2-(2-(Prop-2-yn-1-yloxy)ethoxy)ethyl 4-methylbenzenesulfonate **S2b**. General procedure B was followed using 2-(2-(2-(prop-2-yn-1-yloxy)ethoxy)ethoxy)ethanol **S1b** (3.3 g, 0.020 mol) to give **S2c** as an amber oil (3.1 g, 0.019 mol, 95%). ^{1}H-NMR (300 MHz, $CDCl_3$) δ 2.43–2.45 (m, 4H), 3.56–3.75 (m, 10H), 4.11–4.24 (m, 4H), 7.31–7.38 (m, 2H), 7.74–7.86 (m, 2H). ^{1}H-NMR data were in accordance with the literature [2].

3,6,9,12-Tetraoxapentadec-14-yn-1-yl 4-methylbenzenesulfonate **S2c**. General procedure B was followed using 3,6,9,12-tetraoxapentadec-14-yn-1-ol **S1c** (1.4 g, 5.94 mmol) to give **S2c** as an amber oil (2.9 g, 5.40 mmol, 91%). ^{1}H-NMR (300 MHz, $CDCl_3$) δ 2.42 -2.44 (m, 4H), 3.54–3.75 (m, 14H), 4.08–4.26 (m, 4H), 7.30–7.40 (m, 2H), 7.74–7.86 (m, 2H). ^{1}H-NMR data were in accordance with the literature [5].

3,6,9,12,15-Pentaoxaoctadec-17-yn-1-yl 4-methylbenzenesulfonate **S2d**. General procedure B was followed using 3,6,9,12,15-tetraoxaoctadec-14-yn-1-ol **S1d** (1.8 g, 6.69 mmol) to give **S2d** as an amber oil (2.5 g, 5.75 mmol, 86%). ^{1}H-NMR (300 MHz, $CDCl_3$) δ 2.42–2.45 (m, 4H), 3.55–3.79 (m, 18H), 4.05–4.24 (m, 4H), 7.28–7.39 (m, 2H), 7.74–7.85 (m, 2H). ^{1}H-NMR data were in accordance with the literature [6].

General Procedure C: Synthesis of Azide **S3**

Caution: *The following procedures should be performed with care given the explosive nature of azide-containing compounds*.

tert-Butyl ammonium iodide (10 mol%) was added to solution of the **S2** in anhydrous DMF (50 mL) under a nitrogen atmosphere followed by sodium azide (1.1 eq.). The reaction mixture was then heated at 45 °C for 20 hours before being concentrated in *vacuo* to give a colourless oily solid. The residue obtained was triturated with Et_2O and the insoluble salt removed by filtration. The filtrate was then concentrated in *vacuo* to give a colourless oil, which was taken up in toluene. The mixture obtained was once again concentrated in *vacuo* to give **S3**.

3-(2-(2-Azidoethoxy)ethoxy)prop-1-yne **S3a**. General procedure C was followed using 2-(2-(2-(prop-2-yn-1-yloxy)ethoxy)ethoxy)ethanol **S2a** (5.3 g, 0.020 mol) to give **S3a** as an amber oil (1.6 g, 0.011 mol,

55%). ^{1}H-NMR (300 MHz, $CDCl_3$) δ 2.42–2.45 (m, 1H), 3.35–3.47 (m, 2H), 3.61–3.80 (m, 6H), 4.21–4.24 (m, 2H). ^{1}H-NMR data were in accordance with the literature [7].

3-(2-(2-(2-Azidoethoxy)ethoxy)ethoxy)prop-1-yne **S3b**. General procedure C was followed using 2-(2-(prop-2-yn-1-yloxy)ethoxy)ethyl 4-methylbenzenesulfonate **S2b** (5.5 g, 0.020 mol) to afford **S3b** as an amber oil (2.1 g, 0.012 mol, 61%). ^{1}H-NMR (300 MHz, $CDCl_3$) δ 2.41 -2.43 (m, 1H), 3.34–3.47 (m, 2H), 3.59–3.80 (m, 10H), 4.21–4.23 (m, 2H). ^{1}H-NMR data were in accordance with the literature [2].

1-Azido-3,6,9,12-tetraoxapentadec-14-yne **S3c**. General procedure C was followed using 3,6,9,12-tetraoxapentadec-14-yn-1-yl 4-methylbenzenesulfonate **S2c** (2.0 g, 8.61 mmol) to give **S3c** as a light yellow oil (1.5 g, 5.85 mmol, 68%). ^{1}H-NMR (300 MHz, $CDCl_3$) δ 2.42 -2.44 (m 1H), 3.34–3.46 (m, 2H), 3.60–3.76 (m, 14H), 4.20–4.22 (m, 2H). ^{1}H-NMR data were in accordance with the literature [7].

1-Azido-3,6,9,12,15-pentaoxaoctadec-17-yne **S3d**. General procedure C was followed using **3**,6,9,12,15-pentaoxaoctadec-17-yn-1-yl 4-methylbenzenesulfonate **S2d** (2.4 g, 5.69 mmol) to give **S3d** as a yellow oil (1.5 g, 5.18 mmol, 91%). ^{1}H-NMR (300 MHz, $CDCl_3$) δ 2.42 (t, *J* = 2.4 Hz, 1H), 3.34–3.47 (m, 2H), 3.60–3.79 (m, 18H), 4.20 (d, *J* = 2.3 Hz, 2H). ^{1}H-NMR data were in accordance with the literature [7].

General Procedure D: Staudinger reduction of Azides

Polymer-supported triphenylphosphine (1.15 eq.) was added to a solution of the azido alkyne **S3** in anhydrous THF. The reaction mixture was then stirred at room temperature for 48 days. H_2O (1 mL) was then added to the flask and the resulting mixture stirred at room temperature for a further 48 hours. The reaction mixture was filtered through Celite and the insoluble solid collected washed with EtOAc (25 mL). The filtrate obtained was concentrated *in vacuo* to afford aminoalkyne **6**.

2-(2-(Prop-2-yn-1-yloxy)ethoxy)ethanamine **6a**. General procedure D was followed using 3-(2-(2-azidoethoxy)ethoxy)prop-1-yne **S3a** (1.5 g, 9.22 mmol) to obtain **6a** as an amber oil (1.1 g, 7.93 mmol, 86%). ^{1}H-NMR (300 MHz, $CDCl_3$) δ 2.41-2.44 (m, 1H), 2.80–3.01 (m, 3H), 3.42–3.78 (m, 7H), 4.20 (d, *J* = 2.4, 2H). ^{1}H-NMR data were in accordance with the literature [8].

2-(2-(2-(Prop-2-yn-1-yloxy)ethoxy)ethoxy)ethanamine **6b**. General procedure D was followed using 3-(2-(2-(2-azidoethoxy)ethoxy)ethoxy)prop-1-yne **S3b** (2.0 g, 9.37 mmol) to obtain **6b** as an amber oil (1.6 g, 8.81 mmol, 94%). ^{1}H-NMR (400 MHz, $CDCl_3$) δ 2.37–2.53 (m, 1H), 2.86 (t, *J* = 5.2 Hz, 1H), 3.47–3.53 (m, 1H), 3.58–3.75 (m, 10H), 4.15–4.26 (m, 2H). ^{1}H-NMR data were in accordance with the literature [2].

3,6,9,12-Tetraoxapentadec-14-yn-1-amine **6c**. General procedure D was followed using 1-azido-3,6,9,12-tetraoxapentadec-14-yne **S3c** (1.4 g, 5.60 mmol) to obtain **6c** as an amber oil (1.2 g, 4.98 mmol, 89%). ^{1}H-NMR (300 MHz, $CDCl_3$) δ 1.19–1.22 (m, 1H), 2.42 (q, *J* = 2.2 Hz, 1H), 2.80–2.91 (m, 1H), 3.32–3.79 (m, 14H), 4.20 (d, *J* = 2.4 Hz, 2H). ^{1}H-NMR data were in accordance with the literature [7].

3,6,9,12,15-Pentaoxaoctadec-17-yn-1-amine **6d**. General procedure D was followed using 1-azido-3,6,9,12,15-pentaoxaoctadec-17-yne **S3d** (1.5 g, 4.98 mmol) to obtain **6d** as an amber oil (1.2 g, 4.38 mmol, 88%). ^{1}H-NMR (400 MHz, $CDCl_3$) δ 1.80–1.88 (m, 1H), 2.41–2.44 (m, 1H), 2.81–2.90 (m, 1H), 3.44–3.79 (m, 18H), 4.19 (d, *J* = 2.4 Hz, 2H). ^{1}H-NMR data were in accordance with the literature [7].

Synthesis of aminoazide **7**

Scheme S2. Synthesis of aminoazide **7**.

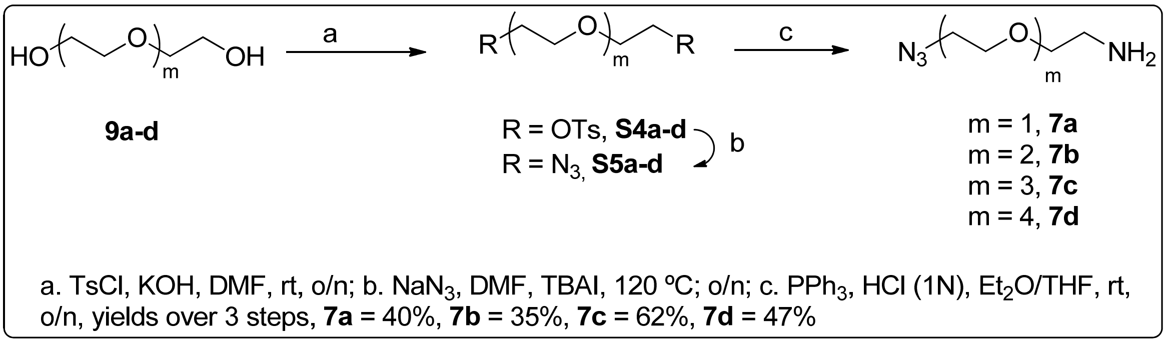

General Procedure E: Di-tosylation of polyethylene glycol

p-Toluene sulfonyl chloride (2 eq.) was added at 0 °C to a solution of the polyethylene glycol **9** in anhydrous DCM. Freshly ground potassium hydroxide (8 eq.) was carefully added in small portion to keep the temperature of the reaction mixture below 5 °C. After complete addition of the base, the reaction was stirred at 0 °C for 3 hours. The mixture was then poured onto ice/water. The 2 layers were separated and the aqueous one extracted with DCM (3 × 25 mL). The combined organic extracts were washed with H_2O (2 × 50 mL), dried over $MgSO_4$, filtered and concentrated in *vacuo* to give **S4**.

Oxybis(ethane-2,1-diyl) bis(4-methylbenzenesulfonate) **S4a**. General procedure E was followed using diethylene glycol **9a** (10 mL, 0.10 mol) to give **S4a** as a colourless oil (25 g, 0.090 mol, 90%). ^{1}H-NMR (400 MHz, $CDCl_3$) δ 2.44 (s, 6H), 3.57–3.64 (m, 4H), 4.05–4.12 (m, 4H), 7.30–7.39 (m, 4H), 7.73–7.83 (m, 4H). ^{1}H-NMR data were in accordance with the literature [9].

(Ethane-1,2-diylbis(oxy))bis(ethane-2,1-diyl) bis(4-methylbenzenesulfonate) **S4b**. General procedure E was followed using triethylene glycol **9b** (15 mL, 0.11 mol) to give **S4b** as a colourless oil (41 g, 0.090 mol, 82%). ^{1}H-NMR (400 MHz, $CDCl_3$) δ 2.43 (s, 6H), 3.51 (s, 4H), 3.60–3.67 (m, 4H), 4.08–4.18 (m, 4H), 7.29–7.37 (m, 4H), 7.73–7.82 (m, 4H). ^{1}H-NMR data were in accordance with the literature [9].

((Oxybis(ethane-2,1-diyl))bis(oxy))bis(ethane-2,1-diyl)bis(4-methylbenzenesulfonate) **S4c**. General procedure E was followed using tetraethylene glycol **9c** (15 mL, 0.090 mol) to give **S4c** as a yellow oil (42 g, 0.088 mol, 98%). ^{1}H-NMR (300 MHz, $CDCl_3$) δ 2.42 (s, 6H), 3.47–3.72 (m, 12H), 4.08–4.18 (m, 4H), 7.27–7.38 (m, 4H), 7.71–7.82 (m, 4H). ^{1}H-NMR data were in accordance with the literature [9].

3,6,9,12-Tetraoxatetradecane-1,14-diyl bis(4-methylbenzenesulfonate) **S4d**. General procedure E was followed using pentaethyleneglycol **9d** (15 mL, 0.070 mol) to give **S4d** as light yellow oil (35 g,

0.064 mol, 92%). ^{1}H-NMR (300 MHz, $CDCl_3$) δ 2.44 (s, 6H), 3.53–3.75 (m, 18H), 4.05–4.20 (m, 4H), 7.29–7.39 (m, 4H), 7.74–7.85 (m, 4H). ^{1}H-NMR data were in accordance with the literature [9].

General Procedure F: Synthesis of diazide

Caution: *The following procedures should be performed with care given the explosive nature of azide-containing compounds*.

tert-Butyl ammonium iodide (10 mol%) was added to solution of the ditosylated ethylene glycol **S4** in anhydrous DMF (50 mL) under a nitrogen atmosphere followed by sodium azide (4 eq.). The reaction mixture was then heated at 80 °C for 20 hours before being concentrated in *vacuo* to give a colourless oily solid. The residue obtained was triturated with Et_2O and the insoluble salt removed by filtration. The filtrate was then concentrated in *vacuo* to give a colourless oil, which was taken up in toluene. The mixture obtained was once again concentrated in *vacuo* to give **S5**.

1-Azido-2-(2-azidoethoxy)ethane **S5a**. General procedure F was followed using oxybis(ethane-2,1-diyl) bis(4-methylbenzenesulfonate) **S4a** (15 g, 0.060 mol) to give **S5a** as a light yellow oil (6.7 g, 0.045 mol, 75%). ^{1}H-NMR (300 MHz, $CDCl_3$) δ 3.41 (t, J = 5.0 Hz, 4H), 3.68 (dd, J = 5.5, 4.5 Hz, 4H). ^{1}H-NMR data were in accordance with the literature [9].

1,2-bis(2-Azidoethoxy)ethane **S5b**. General procedure F was followed using (ethane-1,2-diylbis(oxy))bis(ethane-2,1-diyl) bis(4-methylbenzenesulfonate) **S4b** (25 g, 0.050 mol) to give **S5b** as a light amber oil (8.3 g, 0.035 mol, 70%). ^{1}H-NMR (300 MHz, $CDCl_3$) δ 3.39 (t, J = 5.0 Hz, 4H), 3.58–3.83 (m, 8H). ^{1}H-NMR data were in accordance with the literature [9].

1-Azido-2-(2-(2-(2-azidoethoxy)ethoxy)ethoxy)ethane **S5c**. General procedure F was followed using ((oxybis(ethane-2,1-diyl))bis(oxy))bis(ethane-2,1-diyl)bis(4-methylbenzenesulfonate) **S4c** (25 g, 0.050 mol) to give **S5c** as a light yellow oil (12 g, 0.049 mol, 98%). ^{1}H-NMR (300 MHz, $CDCl_3$) δ 3.33–3.46 (m, 4H), 3.61–3.78 (m, 12H). ^{1}H-NMR data were in accordance with the literature [9].

1,14-Diazido-3,6,9,12-tetraoxatetradecane **S5d**. General procedure F was followed using 3,6,9,12-tetraoxatetradecane-1,14-diyl bis(4-methylbenzenesulfonate) **S4d** (25 g, 0.040 mol) to give **S5d** as a light amber oil (12 g, 0.036 mol, 90%). ^{1}H-NMR (300 MHz, $CDCl_3$) δ 3.39 (dd, J = 5.6, 4.6 Hz, 4H), 3.61–3.75 (m, 16H). ^{1}H-NMR data were in accordance with the literature [9].

General Procedure G: Mono-Staudinger reduction of Azides

A solution of triphenylphosphine in Et_2O was added dropwise over 30 minutes to a solution of the diazido compound **S5** in a mixture of Et_2O/THF/HCl 1N (5/1/5) at room temperature. The reaction mixture was then vigorously stirred at this temperature overnight. The 2 layers were then separated and the aqueous one extracted with DCM (3 × 15 mL). The aqueous layer was then basified by the addition of sodium hydroxide pellets to pH 14. The resulting solution was then extracted with DCM (4 × 25 mL) and the combined organic extracts dried over Na_2SO_4, filtered and concentrated in *vacuo* to give the aminoazide **7**.

2-(2-Azidoethoxy)ethanamine **7a**. General procedure G was followed using 1-azido-2-(2-azidoethoxy)ethane **S5a** (8.0 g, 0.050 mol) to give **7a** as a light yellow oil (3.9 g, 0.029 mol, 59%). ^{1}H-NMR (400 MHz, $CDCl_3$) δ 2.88 (t, *J* = 5.1 Hz, 2H), 3.35–3.42 (m, 2H), 3.49–3.58 (m, 2H), 3.60–3.72 (m, 2H). ^{1}H-NMR data were in accordance with the literature [10].

2-(2-(2-Azidoethoxy)ethoxy)ethanamine **7b**. General procedure G was followed using 1,2-bis(2-azidoethoxy)ethane **S5b** (10 g, 0.040 mol) to give **7b** as a light yellow oil (4.9 g, 0.024 mol, 61%) ^{1}H-NMR (400 MHz, $CDCl_3$) δ 2.82–2.91 (m, 2H), 3.34–3.42 (m, 2H), 3.51 (td, *J* = 5.2, 1.0 Hz, 2H), 3.57–3.71 (m, 6H). ^{1}H-NMR data were in accordance with the literature [11].

2-(2-(2-(2-Azidoethoxy)ethoxy)ethoxy)ethanamine **7c**. General procedure G was followed using 1-azido-2-(2-(2-(2-azidoethoxy)ethoxy)ethoxy) ethane **S5c** (10 g, 0.040 mol) to give **7c** as a light yellow oil (5.8 g, 0.026 mol, 65%). ^{1}H-NMR (400 MHz, $CDCl_3$) δ 2.80 (td, *J* = 5.2, 1.7 Hz, 2H), 3.28–3.36 (m, 2H), 3.45 (m, 2H), 3.51–3.65 (m, 10H). ^{1}H-NMR data were in accordance with the literature [12].

14-Azido-3,6,9,12-tetraoxatetradecan-1-amine **7d**. General procedure G was followed using 1,14-diazido-3,6,9,12-tetraoxatetradecane **S5d** (10 g, 0.030 mol) to give **7d** as a colourless oil (5.2 g, 0.017 mol, 57%). ^{1}H-NMR (400 MHz, $CDCl_3$) δ 2.83–2.91 (m, 2H), 3.35–3.43 (m, 2H), 3.52 (td, *J* = 5.3, 1.2 Hz, 2H), 3.63–3.68 (m, 14H). ^{1}H-NMR data were in accordance with the literature [13].

Sample Availability: Samples of the compounds **6a**–**d** and **7a**–**d** are available from the authors.

^{1}H-NMR Spectrum of ***t*Bu-MTX-Cmpd2.2**

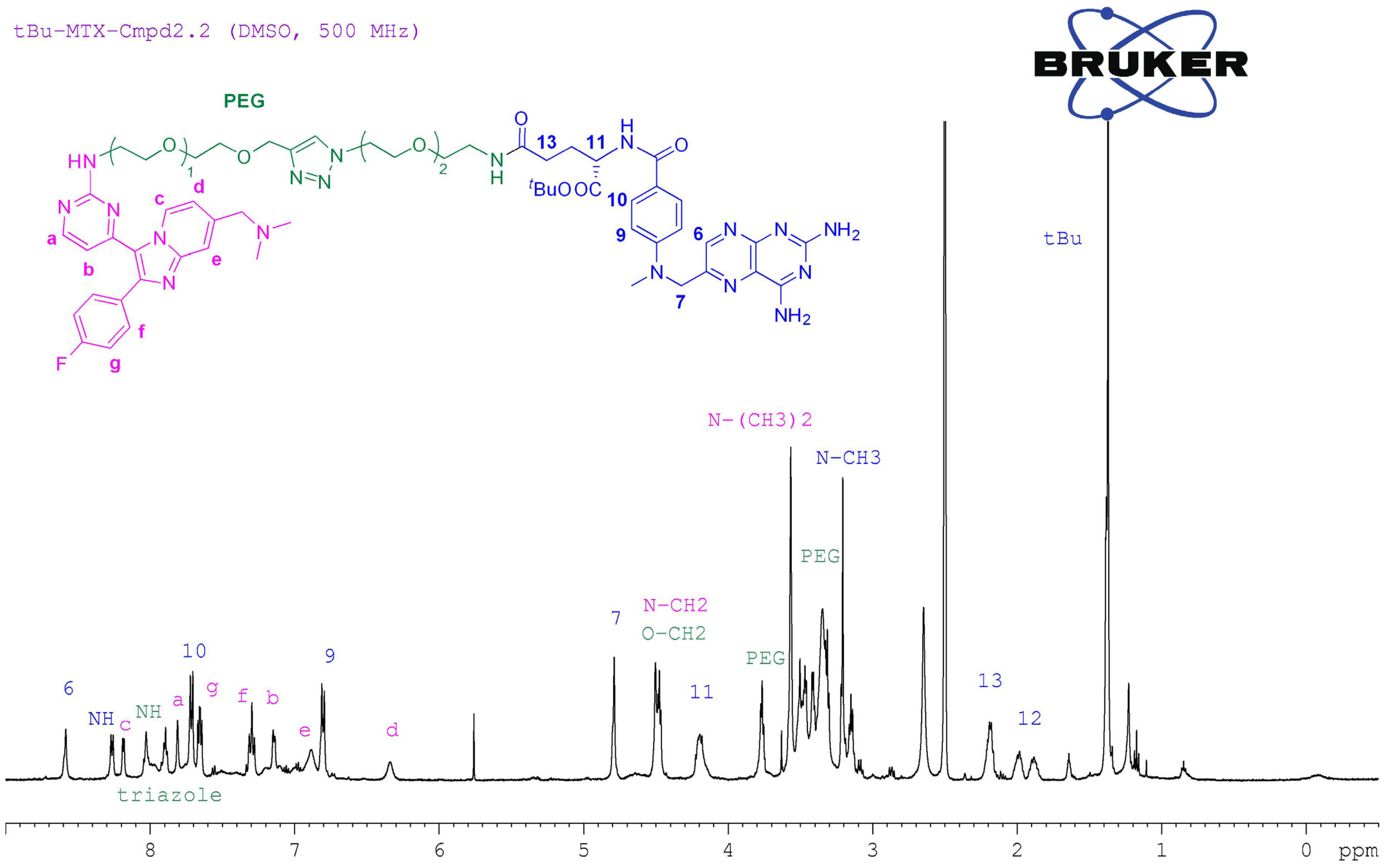

^{13}C-NMR Spectrum of **[t]Bu-MTX-Cmpd2.2**

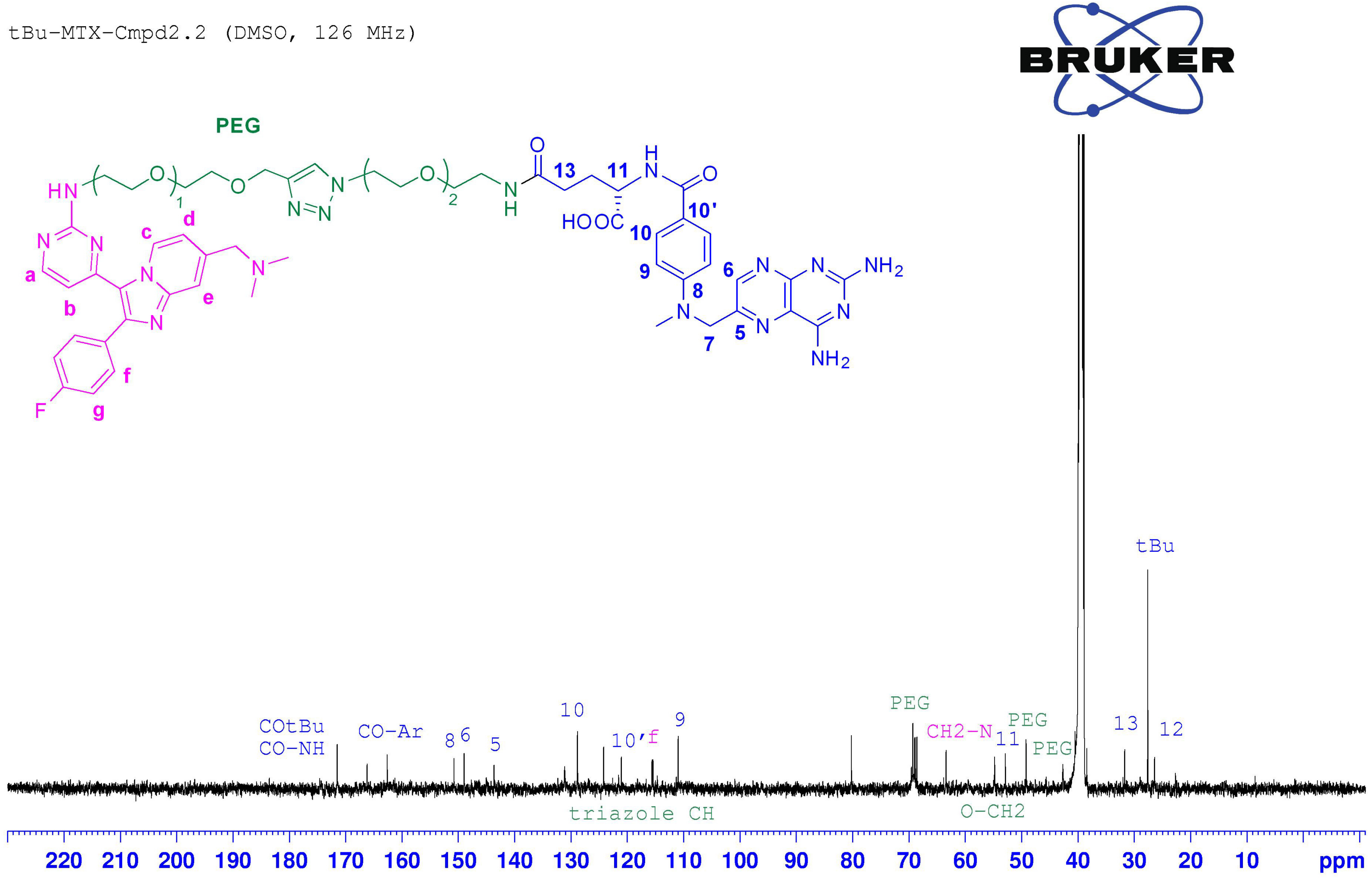

Partial 2D HSQC Spectrum of ^{t}**Bu-MTX-Cmpd2.2** (δH/ δC: 5.90–8.90/102 - 165)

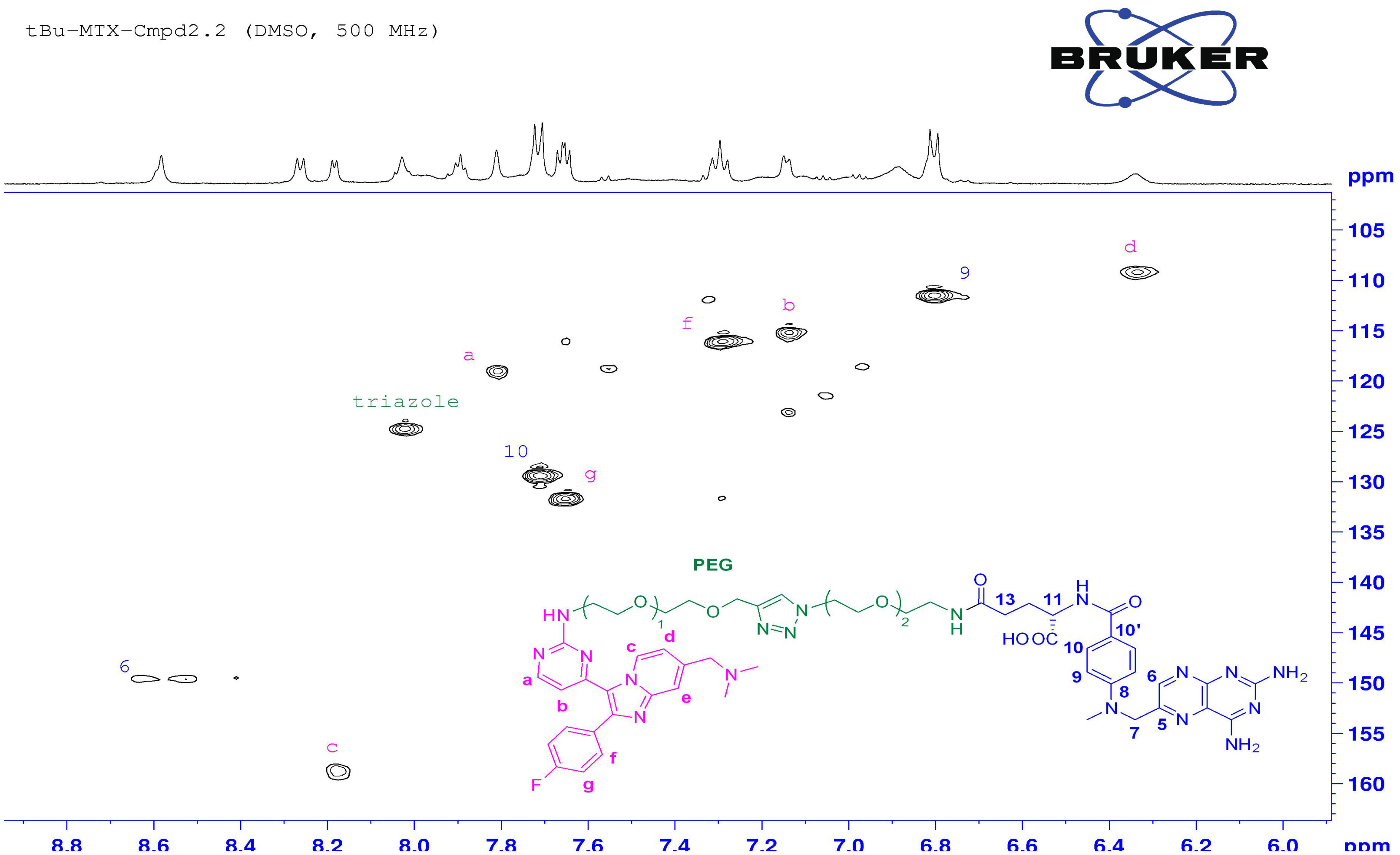

2D HMBC Spectrum of ***t*Bu-MTX-Cmpd2.2**

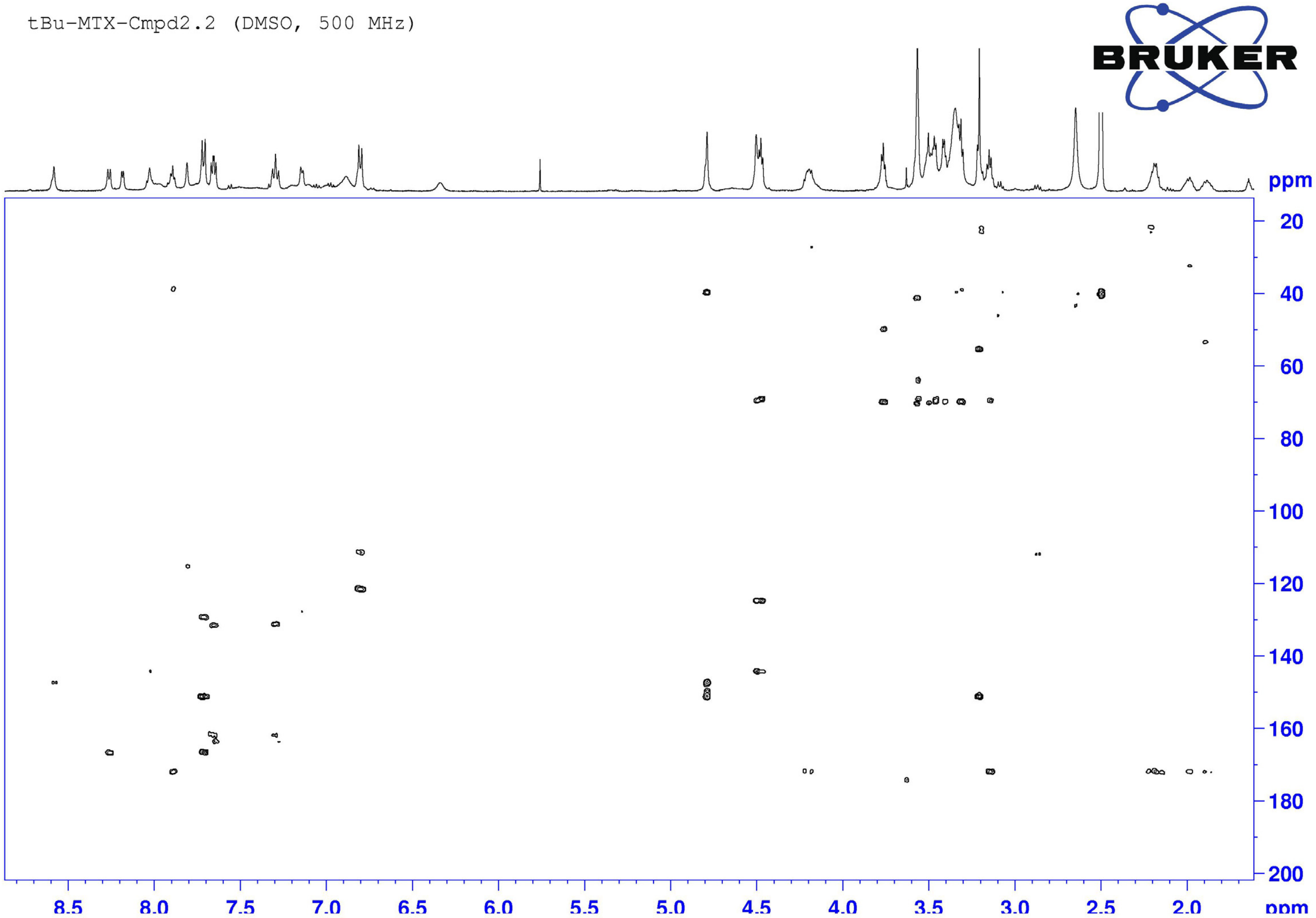

^{1}H-NMR Spectrum of **MTX-Cmpd2.2**

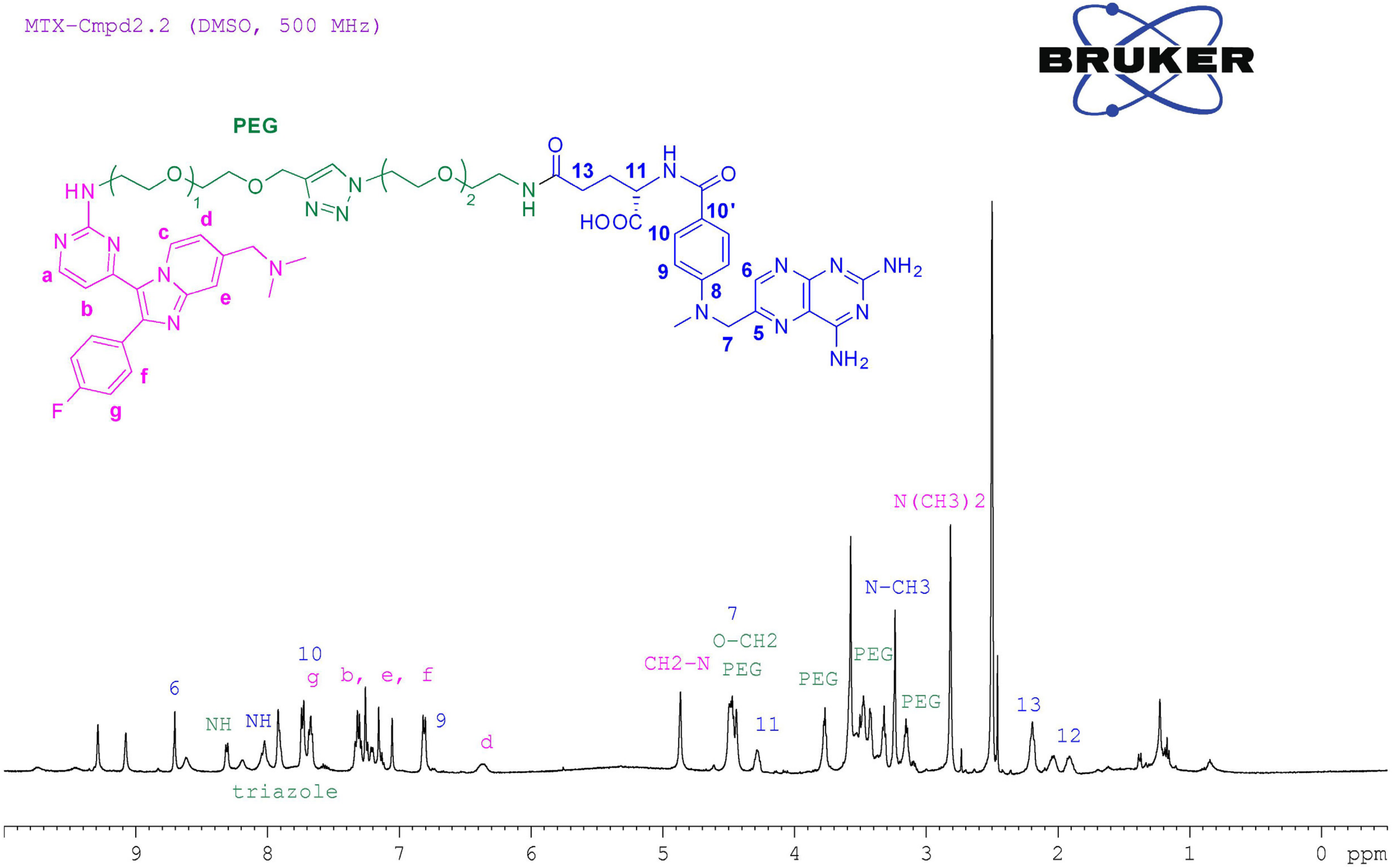

^{13}C-NMR Spectrum of **MTX-Cmpd2.2**

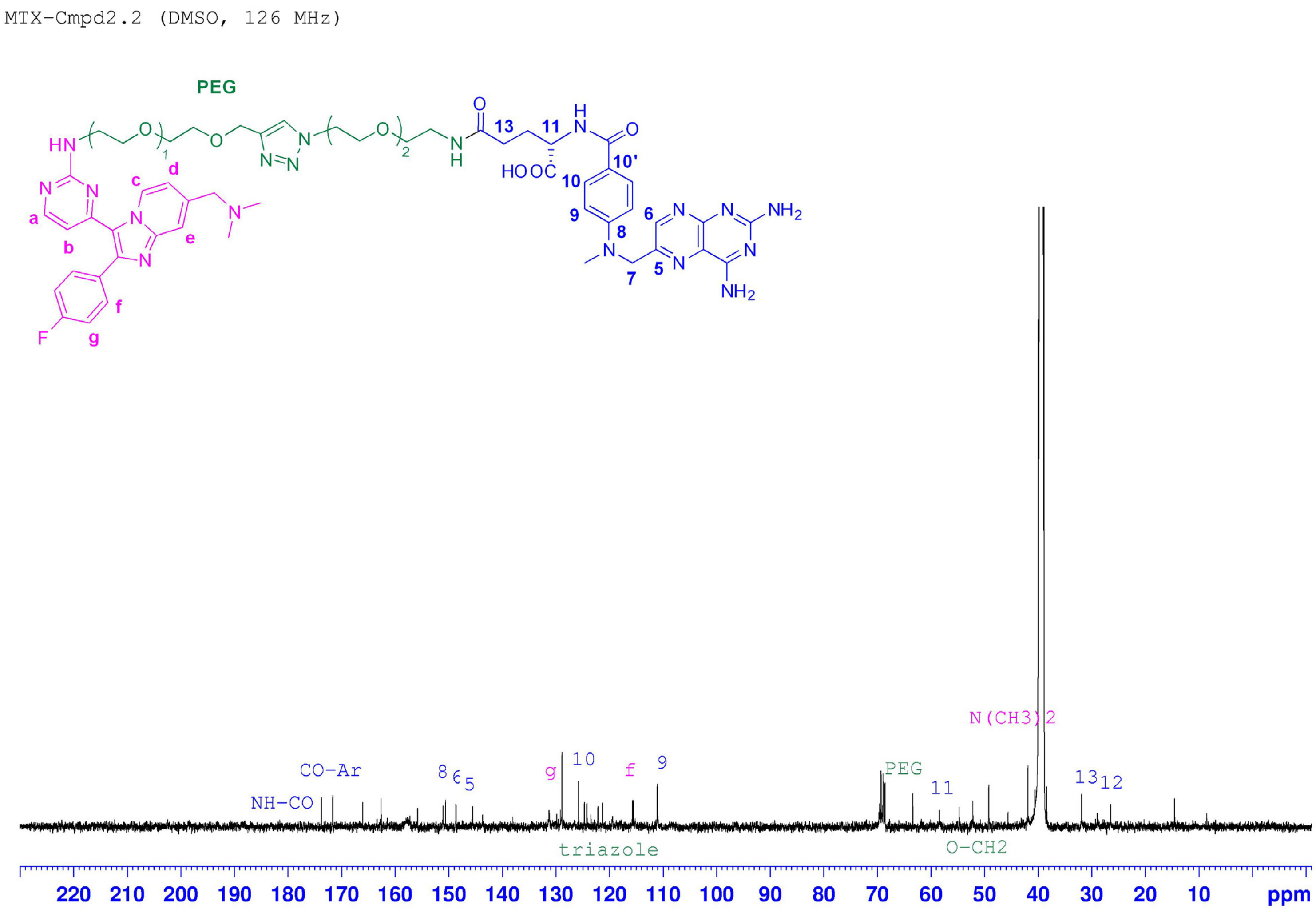

2D HMBC Spectrum of **MTX-Cmpd2.2**

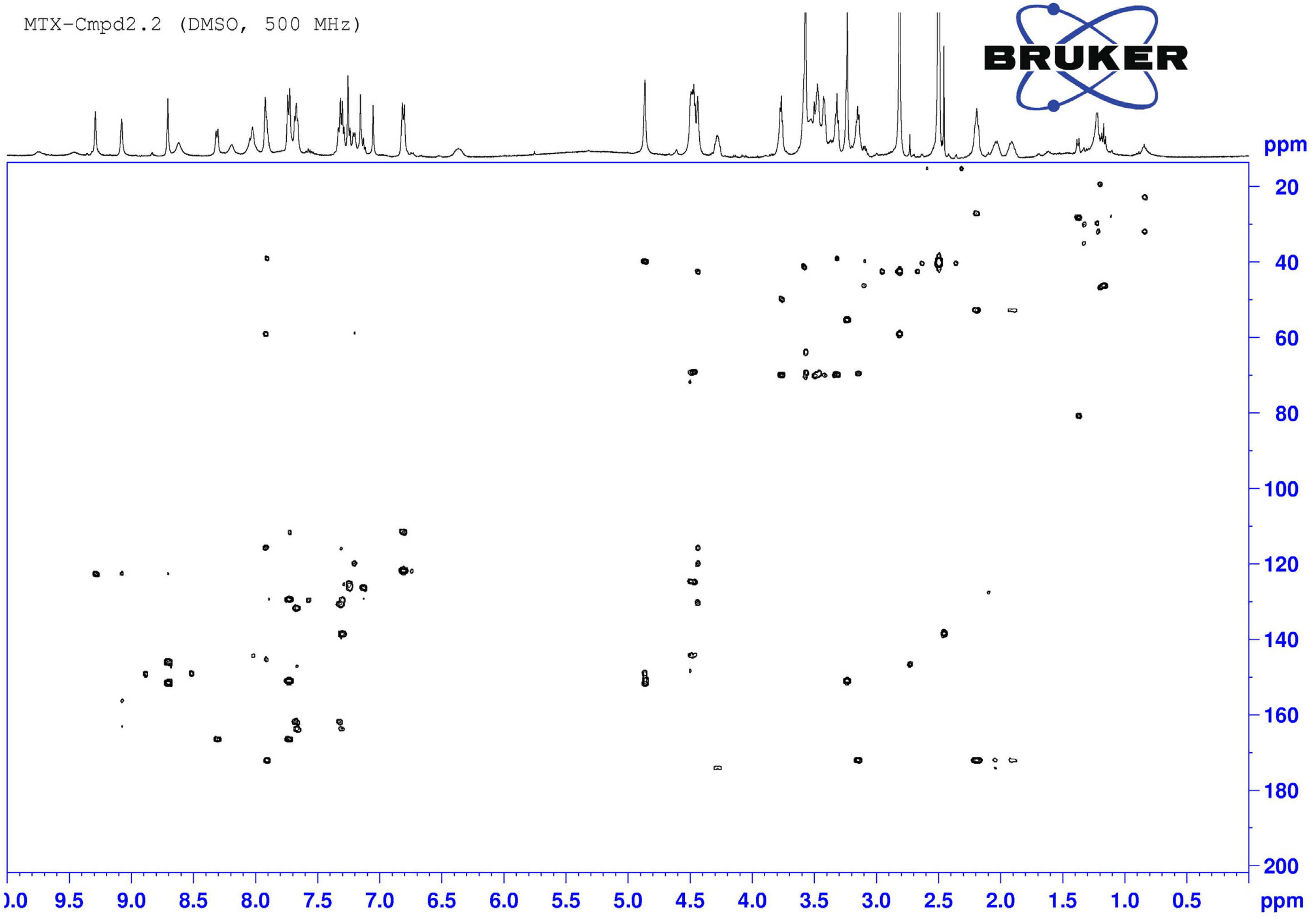

References

1. Gill, H.S.; Tinianow, J.N.; Ogasawara, A.; Flores, J.E.; Vanderbilt, A.N.; Raab, H.; Scheer, J.M.; Vandlen, R.; Williams, S.-P.; Marik, J. A Modular Platform for the Rapid Site-Specific Radiolabeling of Proteins with 18F Exemplified by Quantitative Positron Emission Tomography of Human Epidermal Growth Factor Receptor 2. *J. Med. Chem.* **2009**, *52*, 5816–5825.
2. Norberg, O.; Deng, L.; Yan, M.; Ramstro□m, O. Photo-Click Immobilization of Carbohydrates on Polymeric Surfaces—A Quick Method to Functionalize Surfaces for Biomolecular Recognition Studies. *Bioconjug. Chem.* **2009**, *20*, 2364–2370.
3. Ozdemir, M.S.; Marczak, M.; Bohets, H.; Bonroy, K.; Roymans, D.; Stuyver, L.; Vanhoutte, K.; Pawlak, M.; Bakker, E. A Label-Free Potentiometric Sensor Principle for the Detection of Antibody–Antigen Interactions. *Anal. Chem.* **2013**, *85*, 4770–4776.
4. Diot, J.; Garcia-Moreno, M.I.; Gouin, S.G.; Ortiz Mellet, C.; Haupt, K.; Kovensky, J. Multivalent iminosugars to modulate affinity and selectivity for glycosidases. *Org. Biomol. Chem.* **2009**, *7*, 357–363.
5. Dao, K.-L.; Sawant, R.R.; Hendricks, J.A.; Ronga, V.; Torchilin, V.P.; Hanson, R.N. Design, Synthesis, and Initial Biological Evaluation of a Steroidal Anti-Estrogen–Doxorubicin Bioconjugate for Targeting Estrogen Receptor-Positive Breast Cancer Cells. *Bioconjug. Chem.* **2012**, *23*, 785–795.
6. Zhang, X.; Fang, W.; Mou, T. Pyridazinone with pegylated benzyl triazole as imaging agent and its application. CN 102898421 A, 3 January 2013.
7. Murelli, R.P.; Zhang, A.X.; Michel, J.; Jorgensen, W.L.; Spiegel, D.A. Chemical Control over Immune Recognition: A Class of Antibody-Recruiting Small Molecules That Target Prostate Cancer. *J. Am. Chem. Soc.* **2009**, *131*, 17090–17092.
8. Richards, S.-J.; Jones, M.W.; Hunaban, M.; Haddleton, D.M.; Gibson, M.I. Probing Bacterial-Toxin Inhibition with Synthetic Glycopolymers Prepared by Tandem Post-Polymerization Modification: Role of Linker Length and Carbohydrate Density. *Angew. Chem. Int. Ed.* **2012**, *51*, 7812–7816.
9. Bonger, K.M.; van den Berg, R.J.B.H N.; Heitman, L.H.; Ijzerman, A.P.; Oosterom, J.; Timmers, C.M.; Overkleeft, H.S.; van der Marel, G.A. Synthesis and evaluation of homo-bivalent GnRHR ligands. *Bioorg. Med. Chem.* **2007**, *15*, 4841–4856.
10. Wan, X.; Liu, S. Fabrication of a Thermoresponsive Biohybrid Double Hydrophilic Block Copolymer by a Cofactor Reconstitution Approach. *Macromol. Rapid Commun.* **2010**, *31*, 2070–2076.
11. Klein, E.; DeBonis, S.; Thiede, B.; Skoufias, D.A.; Kozielski, F.; Lebeau, L. New chemical tools for investigating human mitotic kinesin Eg5. *Bioorg. Med. Chem.* **2007**, *15*, 6474–6488.
12. Risseeuw, M.D.P.; De Clercq, D.J.H.; Lievens, S.; Hillaert, U.; Sinnaeve, D.; Van den Broeck, F.; Martins, J.C.; Tavernier, J.; Van Calenbergh, S. A "Clickable" MTX Reagent as a Practical Tool for Profiling Small-Molecule–Intracellular Target Interactions via MASPIT. *ChemMedChem* **2013**, *8*, 521–526.
13. Wen, W.-H.; Lin, M.; Su, C.-Y.; Wang, S.-Y.; Cheng, Y.-S.E.; Fang, J.-M.; Wong, C.-H. Synergistic Effect of Zanamivir−Porphyrin Conjugates on Inhibition of Neuraminidase and Inactivation of Influenza Virus. *J. Med. Chem.* **2009**, *52*, 4903–4910.
